# Supporting the Uptake of Evidence‐Based Care for Degenerative Meniscal Tears and Early Knee Osteoarthritis: A Collaborative Approach to the Development of an Intervention Using Stakeholder Perspectives and Integrating Behaviour Change Theory

**DOI:** 10.1111/hex.70940

**Published:** 2026-11-06

**Authors:** Helen O'Leary, Elaine Toomey, Helen P. French, Liam Glynn, Katie Robinson, Liam G. Ryan, Clodagh Toomey, Hazel Jenkins, Karen McCreesh

**Affiliations:** ^1^ School of Allied Health and Health Research Institute, Faculty of Education and Health Sciences University of Limerick Limerick Ireland; ^2^ Physiotherapy Department University Hospital Kerry Tralee County Kerry Ireland; ^3^ Centre for Health Research Methodology University of Galway Galway Ireland; ^4^ School of Physiotherapy, Royal College of Surgeons in Ireland York Street Dublin Ireland; ^5^ School of Medicine University of Limerick Limerick Ireland; ^6^ Department of Chiropractic, Faculty of Medicine, Health and Human Sciences Macquarie University Sydney NSW Australia

**Keywords:** degenerative meniscal tear, early knee osteoarthritis, evidence‐based care, intervention design, public–patient involvement

## Abstract

**Background:**

Management of a degenerative meniscal tear (DMT) or symptoms consistent with early knee osteoarthritis (OA) frequently deviates from international clinical practice guidelines, which recommend education and exercise as first‐line treatments and strongly advise against arthroscopic partial meniscectomy. There is a need for implementation strategies to support the uptake of evidence‐based care.

**Methods:**

A systematic, four‐step intervention development process was used to guide development of a multi‐faceted implementation intervention targeting both individuals with a DMT or early knee OA and their general practitioners (GPs). The process integrated behavioural theory, stakeholder perspectives and evidence on intervention effectiveness. Target behaviours were identified through a literature review and stakeholder consultation. Previously conducted qualitative interviews with 17 GPs and 11 people with DMT were re‐analysed and mapped to the Theoretical Domains Framework (TDF) to identify behavioural barriers and enablers to the targeted behaviours. Behaviour change techniques (BCTs) and intervention components were then selected to address these determinants. Stakeholder input was incorporated to enhance acceptability and feasibility.

**Results:**

The resulting intervention aims to promote guideline‐concordant management of a DMT and early knee OA. A programme theory to guide implementation was developed to describe hypothesised mechanisms of action, and candidate outcome measures were identified to support future evaluation and assess the mediating mechanisms of behaviour change.

**Conclusion:**

Using a systematic, theory‐informed and stakeholder‐engaged approach, we developed a multi‐faceted intervention to support delivery and uptake of evidence‐based care for DMT and early knee OA. The intervention comprised GP educational workshops and resources to support an ‘enhanced consultation’, alongside referral to an early ‘best practice’ physiotherapy session. Patients received both components. The intervention was designed to be feasible, acceptable and locally relevant, but requires further testing to assess implementation and potential for scale‐up in clinical practice.

**Lived Experience or Public Contribution:**

Stakeholder contributions played a central role in this study. People with lived experience of degenerative meniscal tears and/or knee OA (*n* = 6) and healthcare professional stakeholders (*n* = 15) were consulted during group discussions and individual meetings. Stakeholders informed every step of the development process, aiming to ensure the final intervention was feasible, acceptable and adapted to the local context.

## 1 Background

The burden of knee pain on healthcare resources is increasing [1]. Approximately one in five of all consultations in general practice are musculoskeletal, with the knee being the most common presentation after the lower back [2]. Knee pain also accounts for the greatest number of new consultations seen in Irish secondary care orthopaedic clinics, where meniscal pathology is the most frequent diagnosis after osteoarthritis (OA) [3]. Meniscal pathology in middle‐aged and older adults is usually degenerative, marked by slow progression of the lesion [4]. A degenerative meniscal tear (DMT) in a symptomatic knee is synonymous with early osteoarthritic changes and typically exists in the context of articular cartilage degradation and other early degenerative changes [5, 6]. No clinical tests or symptoms reliably distinguish the two entities, and both symptomatic early‐stage knee OA and a DMT typically present with intermittent, activity‐induced knee pain, with limited or no radiographic changes [7, 8, 9, 10].

Treatment recommendations for DMT and early OA advise a clinical diagnosis without imaging, and conservative management through patient education, exercise therapy and lifestyle change promoting weight management and physical activity [11, 12, 13]. This approach is associated with reduced pain and improved function and quality of life across multiple healthcare settings [14, 15, 16, 17].

Imaging findings have limited relevance to management in early‐stage knee OA [7, 18]. Despite this, inappropriate use of magnetic resonance imaging (MRI) to investigate knee pain in middle‐aged and older adults, without a history of trauma or red flags, is well documented, both in Ireland and internationally [19, 20, 21, 22], and continues to rise [23]. Overuse of diagnostic imaging has been implicated in driving patients with DMT, and their healthcare professionals (HCPs), to seek early orthopaedic or surgical solutions [24, 25, 26], and has been implicated in persistently high knee arthroscopy rates in some countries [26, 27]. Arthroscopic surgery, or partial meniscectomy, as a treatment of DMT is no more effective than sham surgery for improving pain and reducing disability [28, 29], and several studies have associated this procedure with a higher risk of OA progression [30, 31, 32]. Importantly, continued use of ‘unnecessary interventions’ for knee OA, such as MRI and arthroscopy, does not appear to translate into better clinical outcomes [23, 33] and their overuse has important resource implications, further underscoring the need to reduce mismanagement and low‐value care [34, 35].

Meanwhile, there is sub‐optimal use of recommended care for DMT and early OA. The core treatments of education, exercise and weight management are under‐prescribed for knee OA, and there is a low uptake by patients [36, 37, 38, 39, 40]. A substantial proportion of patients with knee OA referred for orthopaedic opinion have not adequately trialled first‐line care, including exercise therapy [41, 42]. Qualitative research with people diagnosed with DMTs and HCPs underscores these findings. GPs and patients describe bypassing first‐line conservative care, such as physiotherapy, in favour of a surgical opinion [24, 25, 43]. Physiotherapists also described a pattern of care that involved unwarranted imaging and early referral to orthopaedic specialists [44].

To bridge the evidence–practice gap, there is a need to identify the HCP, patient and health service barriers to recommended care and address these with a multi‐level intervention. A recent systematic review of the implementation of OA management programmes in primary and community care identified barriers and enablers across individual, service and health‐system levels, including factors related to HCP's knowledge and skills, patient beliefs and capabilities, and limited public funding and workforce [45]. These findings reinforce the need for implementation strategies that address determinants of care at multiple levels rather than focusing solely on professional knowledge or behaviour. The National Institute for Health and Care Research (NIHR) and the Medical Research Council (MRC) recommend that such complex interventions should be grounded in the theoretical basis of behavioural change and respond to underlying behavioural and contextual determinants [46].

Several studies have used theory‐driven approaches in designing interventions to better align knee OA care with best‐practice recommendations by improving the delivery of first‐line care or optimising referral pathways [47, 48, 49, 50]. However, a scoping review of existing models and interventions to improve delivery of non‐surgical OA management in primary care identified important limitations, including insufficient integration of implementation and behavioural change theory and limited stakeholder involvement in intervention development [51].

Another criticism of the existing interventions is their reliance on a ‘one size fits all’ approach despite the highly heterogeneous clinical knee OA population [51]. There is increasing recognition of the importance of focusing on individuals with early symptomatic knee OA and intervening at an earlier stage of disease. This reflects the concept of a ‘window of opportunity’ in early knee OA [8], particularly given evidence that a new diagnosis of knee OA is strongly associated with subsequently developing a range of new comorbidities and functional impairments [52]. To date, most interventions developed to improve evidence‐based care for knee OA have focused on people with an established diagnosis of OA and have typically included both hip and knee OA [47, 53, 54], limiting their applicability to early disease.

While the strong recommendations against surgery for degenerative meniscal pathology are gradually translating to practice [55, 56, 57], uncertainty remains regarding how best to implement conservative management, particularly for people with DMT and early OA. Recent evidence indicates that implementation of recommended OA care is influenced by interacting barriers at patient, HCP, service and health‐system levels [45, 58]. To our knowledge, no theory‐informed intervention with stakeholder involvement has been developed that specifically targets people with DMT and early OA changes and/or their healthcare providers. This study aims to describe the systematic development of a multifaceted implementation intervention to improve the delivery and uptake of evidence‐based care for people with DMTs and early knee OA.

## 2 Methods

### 2.1 Design and Setting

We used a four‐step approach, consisting of guiding questions, first described by French et al., to structure the intervention development process (Figure 1) [59]. This approach combines behavioural change theory, stakeholder involvement and evidence on intervention effectiveness. For further guidance, we also drew on case examples that outlined their use of this four‐step development process [49, 60]. The process is reported in line with the Guidance for the Reporting of Intervention Development (GUIDED) checklist [61]. The Guidance for Reporting Involvement of Patients and the Public (GRIPP) 2 short form (SF) reporting guideline checklist [62] was also followed. Both reporting guidelines are presented in Supplementary File 1. The research team provided expertise in knee OA, general practice, orthopaedics, qualitative, health services research and designing behavioural theory informed interventions to the development process.

**Figure 1:**
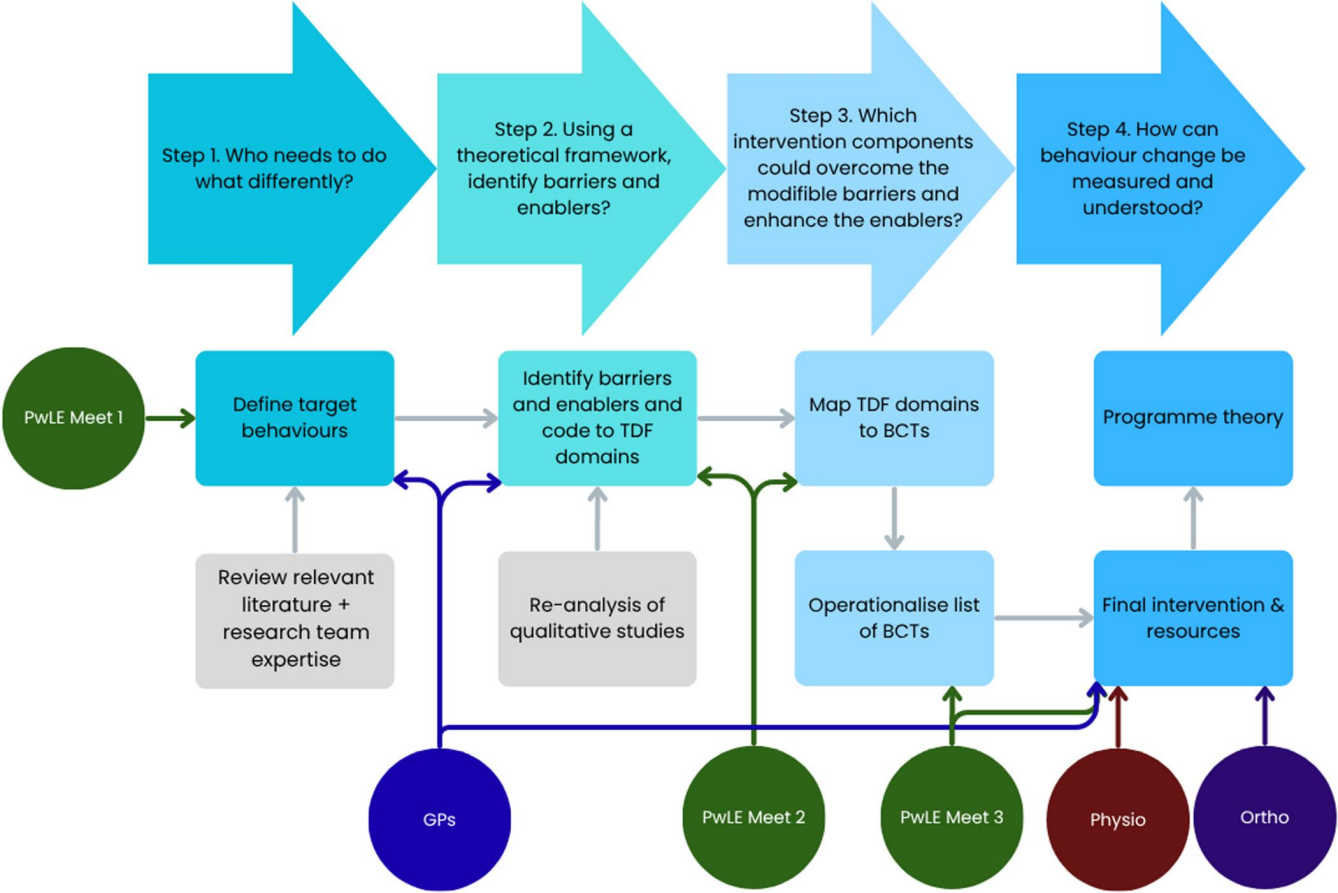
Outline of the four‐step design process by French et al. [59] with timing of stakeholder contributions. Key = BCTs, behavioural change techniques; Meet, stakeholder meeting; Ortho, orthopaedic consultant stakeholders; Physio, physiotherapist stakeholders; PwLE, people with lived experience; TDF, Theoretical Domains Framework.

This intervention development study is part of the Knowledge translation and Exercise for Degenerative Meniscal Pathology and Early osteoarthritis (KNEE‐DEeP) project, which builds on earlier studies [25, 43, 44], with plans to test the intervention in a future KNEE‐DEeP feasibility study [63]. The project is set in the Southwest region of Ireland, where people with knee pain access a mixed public–private system of healthcare. The number of people paying for private health insurance is substantial, estimated to be 48% of the population. Publicly funded physiotherapy is delivered by the Health Services Executive (HSE), but long waiting times can hinder access, and not all patients are entitled to this service [64]. Approximately 42% of the Irish population has access to free GP care [65]. Patients without entitlement to public physiotherapy or free GP care pay out‐of‐pocket [66].

### 2.2 Stakeholder Consultation

Stakeholder consultation is a key component of intervention development, ensuring interventions are acceptable, engaging and feasible prior to piloting [67, 68]. This is particularly important for DMT and early OA, where the evidence base for implementing non‐surgical care remains limited. Stakeholders included HCPs and people with lived experience (PwLE) of DMT and/or early knee OA. All stakeholders were distinct from participants in the baseline qualitative studies.

#### 2.2.1 Stakeholders With Lived Experience of Knee Pain

A panel of six PwLE of DMT/early OA were recruited from the Southwest using an information flyer distributed through physiotherapy clinics and social media. These middle‐aged and older adults represented a spectrum of people with degenerative knee disease who had sought care from the primary and secondary healthcare services in Ireland. Two stakeholders had previously undergone arthroscopy, three had early OA changes or a diagnosis of a DMT and one had established OA.

#### 2.2.2 HCP Stakeholders

These stakeholders, including six general practitioners (GPs) based in the Southwest of Ireland and six physiotherapists working in public musculoskeletal services, were recruited through professional networks. In addition, two orthopaedic surgeons from public hospitals and one health service (physiotherapy) manager were consulted.

#### 2.2.3 Stakeholder Consultation

Stakeholder consultation occurred across the intervention development process. Stakeholder groups were consulted separately to acknowledge potential power dynamics and to facilitate in‐depth discussion within each group. Three in‐person meetings were held with PwLE at early, middle and late stages to inform and refine the intervention. GP input on intervention development was obtained during a group online meeting hosted on Microsoft Teams. During a separate 1‐h meeting, held in‐person, input was sought on intervention feasibility and acceptability from the group of physiotherapist stakeholders. Additional feedback from a subset of these GP and physiotherapist stakeholders was collected via email during finalisation of the intervention. Two orthopaedic surgeons and a health service manager also provided feedback on intervention feasibility and acceptability during one‐to‐one in‐person meetings.

Stakeholder perspectives were considered iteratively at each stage of intervention development by the researchers, when stakeholder groups were invited to consider various aspects of the intervention (e.g., proposed target behaviours, identified barriers and enablers, and draft intervention components). No formal consensus methods were used, and there was no pre‐defined threshold for stakeholder agreement. Areas of agreement and disagreement within and across the different stakeholder groups were identified and discussed by the research team and considered alongside the evidence, behavioural theory and local feasibility.

All consultations were facilitated by a practising physiotherapist and member of the research team (H.O.L). Stakeholders provided written informed consent for audio‐recording of consultation meetings and the use of anonymised extracts. Meeting transcripts were analysed using qualitative content analysis to extract and organise stakeholder feedback that informs the intervention development process. Relevant sections of each transcript were identified and coded according to their content and relevance to intervention development. Codes were grouped into broader categories representing the issues raised by stakeholders. The analysis focused on stakeholder views regarding: (i) the relevance and acceptability of proposed target behaviours; (ii) perceived barriers and enablers to the target behaviours; (iii) preferences for how barriers and enablers could be addressed; (iv) the acceptability, feasibility and practicability of proposed intervention components and modes of delivery; and (v) suggested modifications to proposed intervention content or delivery. Stakeholder groups were considered separately to preserve the perspectives of different groups. Analysis remained primarily focused on the practical implications of stakeholder feedback for intervention development. Findings were summarised for consideration by the research team and used to inform the next step of the intervention development process. Ethical approval for stakeholder consultation was granted by the University of Limerick Faculty of Education and Health Science Research Ethics Committee on 16 February 2023 (Project ID: 2023_02_20_EHS).

### 2.3 Applying a Stepwise Approach to Intervention Development

The approach by French et al. [59] is outlined in the following four steps.

### 2.4 Step 1: Who Needs to Do What, Differently?

Delivering evidence‐based care for DMT and early knee OA requires HCP and patient behaviours to align with best‐practice guidelines. Our focus was on the provision and uptake of core recommended treatments, namely education and exercise, while reducing the use of low‐value care such as inappropriate imaging and referral to secondary care, without first trialling core treatments. Deviations from best practice guidelines were identified through review of the Irish and international DMT and OA literature describing current practice and behaviours [25, 37, 43, 44, 69]. We augmented this with other relevant studies from the knee OA and DMT literature [24, 36, 40].

#### 2.4.1 Step 1 Stakeholder Consultation

In the initial stage, input from GP and PwLE stakeholders helped refine the target behaviours. At the first in‐person meeting, PwLE shared their previous experiences of seeking care and features of an ideal consultation for knee pain. GPs offered suggestions at their online meeting to improve current care and identified behaviours that would be pragmatic and feasible within routine consultations.

### 2.5 Step 2: Using a Theoretical Framework, Which Barriers and Enablers Need to Be Addressed?

To identify specific targets for behaviour change, we applied the refined Theoretical Domains Framework (TDF) (V.2) to explore barriers and enablers to the implementation of recommended behaviours by GPs and patients [70]. The refined TDF comprises 14 domains (Knowledge, Skills, Memory, Attention, and Decision Processes, Behavioural Regulation, Environmental Context and Resources, Social Influences, Beliefs About Capabilities, Social/Professional Role and Identity, Beliefs About Consequences, Emotions, Goals, Intentions, Reinforcement, and Optimism/Pessimism) that influence behaviour and has been effectively used in musculoskeletal research to identify behavioural determinants [49, 71]. Beyond providing a detailed understanding of target behaviours, the TDF also supports the selection of appropriate strategies and techniques aimed at modifying clinical behaviours and improving clinical practice [72, 73].

To understand which barriers and enablers needed to be addressed, we primarily drew on previously conducted qualitative studies with GPs, physiotherapists and individuals with DMT, completed by members of the research team [25, 43, 44]. The analysis was informed by the TDF domain definitions, which were used within a deductive framework to organise barriers and enablers relevant to the target behaviours.

For the GP perspective, qualitative interview data from 17 GPs exploring their management of DMTs were re‐analysed to explore why evidence‐based behaviours were not routinely enacted in clinical practice. The methods and primary findings from this study are reported in detail elsewhere [43]. To understand the perspectives of PwLE, previously collected interview data from 11 individuals with knee pain attributed to DMT, which explored their treatment experiences and expectations, were also re‐analysed [25]. These datasets were selected for secondary analysis because they contained rich accounts from the key participant groups relevant to the target behaviours and participants with direct experience of delivering or receiving care for DMT or early OA within the context of the Irish health service. Although existing literature provides a broader evidence base regarding barriers and enablers to recommended OA care, the selected datasets offered an opportunity to examine these determinants specifically within the target population and healthcare context of the proposed intervention. Sample sizes were determined in the primary studies and were not recalculated for the secondary analysis. Instead, all transcripts from the selected primary studies were reviewed, and each manuscript was found to contain excerpts relevant to the target behaviours, and these were included in the analysis.

For this secondary analysis, a hybrid inductive–deductive approach to qualitative analysis was employed [74]. First, two researchers (H.O.L. and K.M.C.) independently reviewed a subset of transcripts and coded data relevant to the target behaviours. The researchers compared their coding and discussed discrepancies to refine the interpretation and develop an agreed coding framework. A third researcher (K.R.) was available to resolve disagreements if required but was not needed. The resulting coding framework was then applied by H.O.L. to all relevant transcripts, using NVivo 12 to manage the data. Having inductively assigned codes to the data, next the identified codes were deductively mapped to the relevant domains of the refined TDF independently by two authors (H.O.L. and K.M.C.) [75], with discrepancies discussed until consensus was achieved. Barriers and enablers for each target behaviour were subsequently identified within each relevant TDF domain.

TDF domains were considered prominent when they were directly relevant to the target behaviour, expressed by multiple participants, and judged to be likely to influence the target behaviours. Barriers and enablers were prioritised if they were frequently identified and considered potentially modifiable. A subsequent discussion with the core research team (H.O.L., K.M.C., L.R., L.M., C.T. and H.F.) refined and confirmed the TDF domains and determinants. This discussion focused on stakeholder feedback, implications for intervention development within the health service context and the wider OA literature.

#### 2.5.1 Step 2 Stakeholder Consultation

At a second in‐person meeting, PwLE were presented with and discussed a lay summary of the identified barriers and enablers derived from the secondary qualitative data analysis. GP stakeholders were similarly presented with and discussed a summary of the TDF‐informed analysis during their online meeting. Feedback from both stakeholder groups was used in discussions with the core research team to identify which barriers were considered most modifiable and to build consensus on how these could be broadly addressed within the intervention.

### 2.6 Step 3: Which Intervention Components (BCTs and Mode(s) of Delivery) Could Overcome the Modifiable Barriers and Enhance the Enablers?

i. *Mapping TDF domains to appropriate BCTs*
  A behaviour change technique (BCT) is defined as an observable and replicable component designed to change behaviour [76]. For this step, barriers and enablers, categorised by TDF domains, were linked to appropriate BCTs using the Theory and Techniques Tool (available at https://theoryandtechniquetool.humanbehaviourchange.org/tool). This tool maps 74 BCTs from the BCT Taxonomy (BCTTv1) [73], to their corresponding mechanisms of action in the TDF, and indicates the strength of association based on data from a literature synthesis and an expert consensus study [77]. Two research team members (H.O.L. and K.M.C.) met to create an initial list of BCTs. This selection focused on BCTs that had the strongest evidence according to the Theory and Technique Tool. This initial list was narrowed down by the researchers using stakeholder recommendations on how to address the target behaviours, gathered during Step 2 consultations and what was considered feasible within the scope of the current study.
ii. *Developing intervention components and combining them into an acceptable and deliverable intervention*

Next, we operationalised or transformed shortlisted BCTs into potential intervention content (what will actually be delivered) and identified possible modes of delivery (how each BCT would be delivered). Whilst evidence relating specifically to implementing DMT clinical recommendations is lacking, we considered evidence on the effectiveness of implementation strategies in OA or musculoskeletal care [78, 79]. Discussion among the core research team on what was pragmatic and feasible within the project scope was used to arrive at a consensus on a draft implementation intervention that incorporated operationalised BCTs with proposed modes of delivery.

#### 2.6.1 Step 3 Stakeholder Consultation

At the third in‐person meeting, PwLE were presented with the draft intervention, in which several operationalised BCTs were combined into separate intervention components. PwLE were facilitated to discuss the acceptability and practicability of proposed intervention components and how they would work within the local context. Input was also sought on an early draft of an educational leaflet for patients, which integrated responses to information needs identified by PwLE at earlier stakeholder meetings.

Three GPs who had previously participated in the Step 2 online stakeholder meeting provided feedback on the feasibility and acceptability of the draft intervention via email, with particular focus on the components targeting GPs. Six physiotherapists were consulted at this stage through an in‐person meeting, where perspectives were sought on the acceptability and feasibility of the draft intervention, particularly regarding components proposed for delivery by physiotherapists. Two orthopaedic consultants were provided with an overview of the intervention development process and asked to give individual feedback on the acceptability and feasibility of the proposed intervention.

#### 2.6.2 Decision on Final Intervention Using APEASE Criteria

The core research team met to finalise the intervention content, guided by the APEASE (affordability, practicability, effectiveness, acceptability, side effects and equity) criteria [80]. This framework assists researchers in the later stages of intervention development to assess intervention acceptability and feasibility in a thorough and methodical way [80]. PwLE, GP, physiotherapist and orthopaedic consultant stakeholder feedback informed what was acceptable, practical and equitable. We also considered what was feasible to evaluate within a pilot study and routine practice. For effectiveness, we reviewed any evidence from the OA and musculoskeletal literature that supported the shortlisted components. Affordability and sustainability were assessed in the context of Irish public healthcare resources, with input from the manager of the local publicly funded physiotherapy service.

Training materials for HCPs and patient educational resources created by two team members (H.O.L. and K.M.C.) to accompany the final intervention were circulated by email to a subset of GP, physiotherapist and PwLE stakeholders. Their feedback was used to further refine and finalise these resources.

### 2.7 Step 4. How Can Behaviour Change Be Measured and Understood?

In this step, a theory‐informed logic model was developed by the research team representing the programme theory of the intervention. The ‘active ingredients’ of the intervention were specified using BCTs, with hypothesised mechanisms of action used to describe the processes through which these techniques were expected to influence professional and patient behaviour [77, 81]. We also outlined outcome measures designed to capture change in GP and patient behaviour in a future feasibility study set within the context of the Irish health service. This approach was guided by literature on programme theory [82, 83] and the MRC framework for developing and evaluating complex interventions [46, 84, 85].

## 3 Results

### 3.1 Step 1: Identify Who Needs to Do What, Differently

The target behaviours for this intervention were informed by international guidelines and clinical practice recommendations for the management of knee OA and DMTs, which recommend clinical diagnosis without routine imaging [11, 22, 86, 87] and provision of first‐line treatments (education and structured exercise therapy) [11, 13], and strongly advise against arthroscopic partial meniscectomy [11, 12].

Evidence indicates persistent suboptimal provision and uptake of recommended care for DMT and knee OA [25, 36, 37, 40, 44, 51, 88] alongside continued use of unnecessary low‐value interventions such as MRI and arthroscopy [20, 21, 22, 23]. As change is needed at both the HCP and patient level and as GPs in Ireland act as gatekeepers to specialist referral and publicly funded diagnostic imaging [89], GPs were selected as the primary target HCPs for the intervention.

#### 3.1.1 Integrating Stakeholder Feedback on Target Behaviours

Consultation with GP stakeholders highlighted the importance for GPs of protecting their professional autonomy and flexibility to address each patient presentation individually. Accordingly, rather than mandating GPs to avoid specific clinical behaviours (e.g., imaging or secondary care referral), it was considered more acceptable and pragmatic to advise delaying non‐recommended care, at least until patients were provided with a self‐management and exercise programme. Other proposed target behaviours were that GPs would provide patients with a brief exercise recommendation and a brief clinical assessment during the consultation. GP stakeholders supported this, with the understanding that onward referral to physiotherapy would facilitate more comprehensive assessment and tailored exercise prescription.

PwLE also provided feedback on the proposed target behaviours at their first meeting. While views varied regarding the role of imaging in early management, there was general agreement that deferring imaging, particularly MRI, until after provision of an exercise and self‐management programme was acceptable. Patients emphasised the importance of receiving a diagnosis and explanation of symptoms but agreed that a thorough clinical assessment could help meet their need for symptom explanation and validation.

#### 3.1.2 Target Behaviours

Three target behaviours for GPs and one patient target behaviour are specified in Table 1 by describing who, what, when, where and how often each behaviour is to be performed.

**Table 1:** GP and patient behaviours to be targeted by the intervention.

	GP Target Behaviour 1	GP Target Behaviour 2	GP Target Behaviour 3	Patient Target Behaviour
**Who**	GP	GP	GP	Patient (adults > 35 years* presenting to GP with an episode of non‐traumatic knee pain attributable to a DMT or early OA changes on clinical exam)
**What**	Conducts a clinical assessment and provides a diagnosis or explanation for knee pain	Avoids/delays unnecessary imaging** and/or secondary care until they have attended physiotherapy	Provides education and exercise (brief advice + physiotherapy referral)	Actively engages with self‐management and exercise programme
**When**	At the initial consultation	At the initial consultation	At the initial consultation	Following consultation with the GP and physiotherapist
**Where**	Primary care	Primary care	Primary care	Daily life
**How often?**	Once‐off	Once‐off	Once‐off	As agreed with the physiotherapist in the individual self‐management plan

* Imaging (e.g., MRI and X‐ray) is considered unnecessary in the absence of atypical features on exam and no suspicion of alternative diagnosis (e.g., fracture, inflammatory arthritis and malignancy) (11).

** Age cut‐off suggested by the 2016 EESKA Meniscus Consensus (4).

### 3.2 Step 2: Identify the Barriers and Enablers That Need to Be Addressed Using a Theoretical Framework

#### 3.2.1 Barriers and Enablers to GP Target Behaviours

From the 17 qualitative interviews with GPs, we identified the following 10 TDF domains that prominently influenced the GP target behaviours: knowledge, skills, beliefs about capabilities, environmental context and resources, emotion, motivation, beliefs about consequences, professional role and identity, social influences, memory, attention and decision‐making processes (Table 2).

**Table 2:** GP target behaviours mapped to TDF intervention functions and BCTs.

Target behaviour 1. GP provides the patient with a diagnosis based on clinical history and physical examination			
Barriers	Illustrative quote*	TDF domain	Selected BCTs [73] (*operationalised with mode of delivery)*
Time pressures: Limited consultation time reduces the likelihood of the MSK exam and increases reliance on MRI	*‘Time is often limited, particularly when the knee pain is tagged onto a consultation about another medical matter’*	Environmental context and resources	**1.3 Time management** (in *workshop teach GPs to conduct a focused knee exam in* < *5 min)* **12.5 Adding objects to the environment** + **11.3 Conserving mental resources** (*provision of a quick reference guide to aid diagnosis during GP consultation*)
Low confidence in MSK skills: Uncertainty about the MSK exam	*‘I never did an orthopaedic rotation and yet here I am, the nuts and bolts are back pain, knee pain, hip pain’*	Belief about capabilities; Skills	**6.1 Demonstration of the behaviour** + **4.1 Instruction on how to perform the behaviour** *(workshop demonstration and step‐by‐step guidance on conducting a focused knee exam)* **15.1 Verbal persuasion about capability** (*during workshop, reinforce to GPs that their clinical assessment is sufficient for diagnosis*) **15.3 Focus on past success** *(feedback to GP on discharge summary from physio re‐enforces concordance of GP diagnosis with physio assessment)*
Knee pain is not a priority: Consultation focuses on other comorbidities	*‘Always in real life, you've four problems that the patient will come in with’*	Memory, attention and decision‐making processes	**11.3 Conserving mental resources** (*quick reference guideline to aid GP diagnosis)* **7.1 Prompts/cues** *(checklist embedded in physio referral form to cue GP to provide clinical exam and diagnosis)*
Fear of misdiagnosis and litigation: Anxiety about missing serious pathology	*‘But you're always worried, aren't you? About that one thing that you didn't MRI 10 years ago?’*	Belief about consequences	**5.1 Information about health consequences** + **5.6 Information about emotional consequences** (*workshop education on red flags, emphasising low risk of serious pathology in the absence of these and present diagnosis via clinical exam as safe and evidence‐based care)*

Target Behaviour 2: GP defers or avoids non‐recommended care (imaging and early referral to specialist care) at least until the patient has accessed physiotherapy			
**Barriers and enablers**	Illustrative quote*	**TDF domain**	**Selected BCTs** *(operationalised with mode of delivery)*
Patient expectations and fears of damaging therapeutic relationship: GPs concede to requests for an MRI to avoid conflict	*‘No, I wouldn't fall out with them over it. So, yeah, if people come in demanding an MRI, I just throw my eyes up to heaven to myself, and make the request’*	Social influences; Emotion	**6.3 Information about others' approval** (*during GP workshop, share feedback from orthopaedic consultant and physio stakeholders that MRI is not necessary for evidence‐based care)* **6.1 Demonstration of the behaviour** (*demonstration in GP workshop of how to confidently explain why imaging/referral may be unhelpful and promote use of collaborative approach with patient to enhance buy‐in around deferral of imaging)* **11.2 Reduce negative emotions** (*target GP fear of damaging the patient relationship by reframing clinical exam as a means of reassuring patient and imaging as something that may increase patient worry)*
Desire for a specialist opinion to direct management: Uncertainty about indications for exercise or surgery	*‘We understand a lot of the time, when we do refer to orthopaedics that it may or may not be needed but you want that specialist opinion from secondary care’*	Knowledge; Belief about capabilities	**5.1 Information about health consequences** (*provide evidence‐based infographic illustrating clinical indications for arthroscopy and potential for poorer long‐term outcomes)* **4.1 Instruction on how to perform the behaviour** + **6.1. Demonstration of behaviour** *(in workshop, provide specific guidance on exercise and education, i.e., what GPs should do instead of referring, also provide practical instruction on how GPs can promote physiotherapy intervention as ‘specialist care’)* **10.4 Social reward** (*arrange for positive feedback on discharge summary from physio praising GP's patient selection and re‐enforcing their suitability for non‐surgical management)*
Long wait to access secondary care orthopaedic specialist and lack of other management options: Early referral to orthopaedics used as a workaround	*‘When you refer them, you probably refer them earlier, even if they mightn't need to be referred. Because in your own head, you know they're going to be years waiting, you don't want to waste time’*	Environmental context and resources	**12.2 Restructuring physical environment** (*restructure the care pathway by facilitating early access to ‘best practice’ physiotherapy session as a viable alternative)* **12.5 Adding objects to the environment** (*provide GP with Patient Advice Leaflet containing simple starter exercises and links to exercise videos to support exercise prescription)*
Awareness of the potentially negative consequences of imaging (enabler)	*‘Unfortunately, then it (MRI report) comes back with all this noise around it, which says that things aren't fine. Then the patient is very distressed when you're explaining about the bones and osteophytes’*	Beliefs about consequences	**5.1 Information about health consequences** *(in GP workshop, present evidence to reinforce existing beliefs about potential negative imaging consequences)*
Confidence in the ability to leverage existing trusting relationship with patient to delay non‐recommended care (enabler)	*‘Absolutely, when they trust you, you have the option of “Look we will see how this goes and come back in a couple of weeks and we will review it again”’*	Belief about capabilities	**15.3 Focus on past success** *(in GP workshop, prompt reflection on previous patient encounters where trust enabled delayed action)*

Target Behaviour 3. GP provides the patient with the recommended first‐line treatment of education and exercise			
**Barriers and enablers**	Illustrative quote*	**TDF domain**	**Selected BCTs** *(operationalised with mode of delivery)*
GPs perceive patients to be disinterested in exercise and reluctant to engage with exercise‐based care	*‘I do think there's some bit of reluctance for people to go for physio sometimes. They want a quicker fix’*	Social influences	**6.3 Providing information about others' approval** *(in workshop, provide GPs with sample quotes from PwLE illustrating desire for self‐management strategies and preference to avoid surgery)*
Lack of GP skills and confidence in providing education and exercise	*‘I've neither the time, knowledge or expertise to advise people on this’*	Beliefs about capabilities; Skills	**15.1 Verbal persuasion about capabilities** *(during workshop, reassure GPs that prescribing brief and simple exercise advice is beneficial)* **4.1 Instruction on how to perform a behaviour** + **6.1 Demonstration of behaviour** *(provide instruction on how to deliver basic exercise advice to patients, demonstrate knee exercises during workshop that can be safely prescribed to patients and provide simple educational messages to be delivered verbally by GP)*
Exercise prescription is perceived as the physiotherapist's domain and beyond the GP's remit	*‘I would never recommend anything myself because it's not my area of expertise’*	Professional role and identity	**13.1 Identify associated with changed behaviour** + **9.1 Credible source** (*emphasise exercise prescription as part of GP role in initiating effective, guideline‐based care for knee pain)* **3.1 Social support** (share information from physiotherapist stakeholders that GPs recommending exercise/physical activity for knee pain is professionally valued by physiotherapists)
Lack of resources to support patient education and exercise prescription	*‘I find it hard to find resources online for patients that are consistent, something that is practical, an entry point to low level “Let's get started” pain management and strengthening exercises’*	Environmental context and resources	**12.5 Adding objects to the environment** (*provide GP with Patient Advice Leaflet containing simple starter exercises and links to exercise videos to support exercise prescription)*
Limited access to publicly funded physiotherapy services and the prohibitive cost of private physiotherapy	*‘The problem however with the physio is that with the public service, the waiting list at the moment, I think it's four to six months’*	Environmental context and resources	**12.2 Restructuring physical environment** (*restructure the care pathway by facilitating early access to ‘best practice’ physio session as a viable alternative)*
GPs value physical activity as part of management (enabler)	*‘The emphasis for me, is to keep them active. I think if I can keep them active a lot of things resolve, you know’*	Beliefs about consequences	**5.1 Information about health consequences** *(in GP workshop, present evidence to reinforce existing beliefs about the benefits of exercise)*
Feelings of frustration and guilt with available treatment options (enabler)	*‘The people who aren't bad enough to warrant any surgical intervention. but they feel they can't exercise. You're stuck in the middle here saying, “What else can we do here?”’*	Emotion	**5.6 Information about emotional consequences** *(reframe education and exercise as a constructive action that GP can take and help reduce professional discomfort with limited options)*

Abbreviations: BCT, behavioural change technique; GP, general practitioner; MRI, magnetic resonance imaging; MSK, musculoskeletal; PwLE, people with lived experience; TDF, Theoretical Domains Framework.

* Quote from GP interview study (43).

Barriers to **Target Behaviour 1** (conducts a clinical assessment and provides a diagnosis) included fear of misdiagnosis, low confidence in musculoskeletal skills and competing priorities in a time‐pressurised consultation, which represented a combination of cognitive, emotional and contextual influences as described by the TDF.

Barriers to **Target Behaviour 2** (avoids/defers imaging and referral to secondary care) included managing patient expectations, desire for a specialist opinion (e.g., orthopaedic) and lack of access to other management options such as physiotherapy. Enablers of this target behaviour were GPs' awareness of the negative implications of unnecessary imaging and the leveraging of existing patient relationships to help manage patient expectations.

For **Target Behaviour 3** (provision of education and exercise), GPs' perception of their professional role, perceived patient disinterest, GPs' knowledge and skills, and lack of access to physiotherapy and trustworthy patient educational resources were all cited as barriers. Enablers of this behaviour include GPs' motivation to promote physical activity with their patients and frustration with current management options.

#### 3.2.2 Barriers and Enablers to Patient Target Behaviour

The integration of qualitative data from 11 patient interviews identified barriers and enablers that corresponded to 10 TDF domains (beliefs about consequences, intention, behavioural regulation, motivation, knowledge, belief about capabilities, emotion, social influences, environmental context and resources, and reinforcement) (Table 3).

**Table 3:** Patient target behaviour (engages with non‐surgical management for their knee pain following consultation with the GP and physiotherapist) mapped to TDF intervention functions and BCTs.

Barriers and enablers	Illustrative quote*	TDF domain	Selected BCTs [73] *(operationalised with mode of delivery)*
Negative expectations of conservative management: Surgery perceived as necessary	‘*No, I was advised you need to get that problem sorted, physiotherapy won't do nothing for that, it is still going to be there the following week’*	Beliefs about consequences	**5.1 Information about health consequences** + **13.2 Framing/reframing** + **12.5 Adding objects to the environment** *(GP and physio provide consistent messages on expected positive outcome with conservative management, backed up with information in Patient Advice Leaflet)*
Poor engagement and adherence with exercise/physical activity: Rehabilitation is perceived as a more difficult treatment option	*‘(Surgery is) the easy option if it works. You don't have to be minding the knees and doing the conditioning for them’*	Intentions; Behavioural regulation	**5.1 Information about health consequences** *(patient informed by GP and physio about potential negative consequences of arthroscopy and benefits of exercise for long‐term knee and general health)* **1.3 Goal setting (outcome)** + **1.2 Problem solving** + **1.4 Action planning** *(patient identifies valued functional goals with physio and creates an action plan linking exercise and physical activity to goals)* **2.3 Self‐monitoring of behaviour** *(patient uses smartphone app or paper diary to track exercise/physical activity)*
The ability to exercise (or be physically active) is hampered by a lack of knowledge and confidence	*‘I was a keen cycle enthusiast up until a few months ago, but when I got the results of the MRI, I said I'd better stay off the bike until I get this sorted. It was in my mind, if I keep this up (cycling) would I make it worse? Would I do more damage than good?’*	Belief about capabilities; Knowledge	**8.7 Graded tasks** *(physio provides a graded exercise plan to gradually build confidence)* **15.1 Verbal persuasion about capability** + **15.3 Focus on past success** *(physio explicitly expresses confidence in patient's ability and highlights examples of successfully engaging in physical activities, e.g., cycling)* **4.1 Instruction on how to perform the behaviour** + **8.1 Behavioural practice/rehearsal** *(provision of verbal, written and visual exercise instructions, exercises practised with a physiotherapist)*
Fearful of doing harm with exercise	*‘In the situation where I was afraid to do any exercise, absolutely petrified, until I saw the orthopaedic consultant’*	Emotion	**11.2 Reduce negative emotions** + **13.2 Framing/reframing** *(physio helps patient change interpretation of knee pain from ‘hurt equals harm’ and introduces pain‐monitoring tool to help interpret mild pain/discomfort during exercise more positively)*
Inadequate access to physiotherapy or access to non‐evidence‐based physiotherapy	‘*they (sports team physios) only just advised you would be better getting it sorted surgically first and then build it back up after that’*	Environmental context and resources	**12.1 Restructuring physical environment** *(restructure the care pathway by facilitating early access to ‘best practice’ physiotherapy session)* **12.5 Adding objects to the environment** *(GP provides interim exercises and self‐management advice through a printed exercise programme and exercise videos)*
The perception that a structural diagnosis (using MRI) is essential before treatment	‘*So with the MRI I just wanted to know what's wrong and can it be fixed, that's all’*	Beliefs about consequences	**13.2 Framing/reframing** *(clinical exam is used to reassure [e.g., draw attention to well‐preserved range of motion] and reduce patient fears of serious structural damage)* **5.1 Information about health consequences** + **9.1 Credible source** *(both GP and physio emphasise that starting exercise/physical activity without an MRI is safe, message backed up with Patient Information Leaflet endorsed by academics)*
Reinforcement of MRI/surgery beliefs by others	‘*I just think it has to be done (surgery), because anyone I speak to, and I have an aunt who is a nurse, who was telling me that if I don't look after it now, it will cause a lot more problems down the line’*	Social influences	**6.3 Information about others' approval** (*GP and physio deliver consistent message that important others [experts and clinical guidelines] recommend clinical diagnosis, exercise and self‐management behaviours)*
Positive past experiences with exercise (enabler)	‘*I don't know what it came down to, the improvements, but it seemed it was the more exercise I was doing the better it seemed to get’*	Reinforcement	**10.8 Incentive outcome** *(physio draws attention to the patient's past successes with exercise and accompanying benefits that the patient can attain again)*
Strong desire to return to valued activities (enabler)	‘*My main thing is … just to get it right and to be able to go back to what I used to do’*	Motivation	**1.3 Goal setting (outcome)** *(physio frames goals of rehabilitation around return to the patient's meaningful activities)*

Abbreviations: BCT, behavioural change technique; GP, general practitioner; MRI, magnetic resonance imaging; MSK, musculoskeletal; physio, physiotherapist; PwLE, people with lived experience of knee pain; TDF, Theoretical Domains Framework.

* Quote from an interview study with people with degenerative meniscal tears (25).

Barriers to patient engagement with exercise therapy and self‐management of their knee pain included a lack of knowledge, confidence and fear of doing harm with exercise, negative expectations of conservative management and the perception of surgery as a more straightforward solution. Reinforcement of beliefs about the need for imaging or surgery by others and lack of access to evidence‐based physiotherapy were other barriers to patients engaging with exercise therapy. Enablers included patients' positive past experiences with exercise and their desire to return to valued activities.

### 3.3 Step 3: Which Intervention Components (BCTs and Mode(s) of Delivery) Could Overcome the Modifiable Barriers and Enhance the Enablers?

i. **Identify potential behavioural change techniques and modes of delivery to overcome barriers and enhance the enablers**
  The initial longlist of BCTs mapped to each relevant domain for GPs and patient target behaviours is displayed in Supplementary File 2: Tables A and B. A summary of the rationale for excluding BCTs is summarised in Supplementary File 2: Tables C and D.
ii. **Identify what is likely to be feasible, locally relevant and acceptable and combine identified components into an acceptable intervention that can be delivered**
  The draft intervention, incorporating shortlisted and operationalised BCTs (italicised), stakeholder feedback, and evidence from relevant literature, is outlined below.

#### 3.3.1 Draft Intervention

In musculoskeletal care implementation research, multi‐facet component interventions are more effective than single‐component interventions at changing health professionals' clinical behaviours [79]. Interventions that have demonstrated a small but positive effect on clinical behaviours include dissemination of clinical practice guidelines, interactive educational meetings, monitoring the performance of healthcare delivery, peer assessment, and use of local opinion leaders [79, 90].

Interventions to **enhance the initial GP–patient consultation** that typically involves education and training of the provider (GP) have demonstrated improved uptake of evidence‐based care [50, 91] and, in some instances, reduced low‐value healthcare usage [92], although evidence of improved clinical outcomes compared to usual care is mixed [51]. Such approaches frequently encompass a tailored care plan, delivered by another member of the multi‐disciplinary team [48, 50]. Based on this evidence, our intervention proposed a similar model, with the physiotherapist tailoring a plan to support patients with behavioural change and self‐management.

**Interactive educational workshops** for clinicians have enhanced evidence‐based clinical behaviours for low back pain and reduced low‐value imaging [78, 93] and improved the uptake of core recommended treatments among patients with OA [94]. For workshop content, GP stakeholders expressed interest in understanding the evidence base for management of DMTs and knee OA (*information provision, information about health consequences and credible source)*, alongside acquiring practical assessment and communication skills (*rehearsal of relevant skills, instruction on how to perform a behaviour, and demonstration of a behaviour*). One GP noted:

I think if we have a real good spiel on how to explain or an explanation on when to do an MRI and when not to, that's something that's very helpful

GP Stakeholder 3

In addition to revising practical assessment skills, GPs agreed that a laminated Quick Reference Guide for their consultation room would help aid diagnosis (*conserving mental resource and adding objects to the environment)*. GPs favoured methods to deliver patient education that were quick, simple and therefore likely to be feasible in routine practice.

If you are going to provide something for GPs, it must be, you know, probably no more than three points. “Here is what you say.”

GP Stakeholder 1

In response, **four key patient education messages** for verbal delivery by GPs were identified to support efficient education (*information provision and credible source*). These messages addressed common patient misconceptions and unhelpful beliefs identified in the OA and DMT qualitative data [25, 43, 95]. Supportive, evidence‐based educational messages within ‘enhanced’ GP consultations have improved concordance with low back pain and OA guideline recommendations [96, 97]. PwLE stakeholders emphasised the importance of having this verbal information backed up in a written format (*adding objects to the environment*).

It's having that key information in front of you, and for me personally, I think it has to be in writing.

PwLE Stakeholder 2

Both GP and PwLE stakeholder groups requested a Patient Advice Leaflet containing educational information written in lay language and should include the credentials of the researchers who compiled the information and the project funder (*credible source*). To support exercise provision, GPs requested a simple ‘starter’ for inclusion in the Patient Advice Leaflet, with images and links to videos (*instructions on how to perform behaviour*).

But it would be really nice to have something very quick that you could kind of get them (the patient) started with.

GP Stakeholder 5

The suggestion of a GP follow‐up appointment by PwLE stakeholders was deemed unfeasible by GPs due to time constraints. Instead, it was agreed that a management plan be documented on the Patient Advice Leaflet and include a planned GP follow‐up appointment if necessary.

Several GP stakeholders reiterated the challenge of prioritising knee pain within a multimorbidity consultation:

It's rare enough in my experience that a patient will come and say that my only problem today is knee pain.

GP Stakeholder 6

In response, a checklist embedded in the physiotherapy referral form (*Prompts/cues*) would cue GPs to complete elements of the enhanced consultation, e.g., provide Patient Advice Leaflet. The checklist would also provide repeated reinforcement of desirable behaviours, and successful implementation interventions from low back pain research suggest that at least one component of an implementation intervention should be ongoing over time [98].

Physiotherapist stakeholders supported the initiation of exercise prescription by GPs, noting that it would provide useful preliminary information on patients' responses to exercise. As part of their ‘enhanced consultation’, GPs would refer patients to an early ‘best practice’ physiotherapy session (*Restructuring the social environment*) for tailored advice, exercise prescription and a self‐management plan. The use of a single physiotherapy session was supported by evidence from other chronic musculoskeletal conditions. A robust single session had comparable results for pain, function and quality of life when compared to multiple physiotherapy sessions [99, 100]. To optimise the acceptability of a single session, PwLE stakeholders suggested including access to follow‐up advice over the telephone, where additional guidance was required.

Several self‐regulatory BCTs, which have been shown to boost self‐efficacy and exercise adherence, were integrated into the ‘best practice’ physiotherapy session (*Goal setting [outcome], action planning, self‐monitoring of behaviour, behavioural practice/rehearsal, and adding objects to the environment*) [101, 102, 103]. There was unanimous support from PwLE for the provision of resources and practical strategies to support self‐management (*knowledge and skills and verbal persuasion about capability)*. One stakeholder commented:

I think it's only when the emphasis is put on your own participation in your recovery and in plain simple terms you are told, “You have to do this. and here's how you are going to do it.”

PwLE Stakeholder 4

Physiotherapist stakeholders emphasised the need for a longer session to facilitate the delivery of individualised education and allow time to explore beliefs and expectations related to symptoms and exercise *(reduce negative emotions, re‐attribution, framing/reframing and verbal persuasion about capability)*. Training and resources to support the delivery of this single session were also considered important by physiotherapist stakeholders. Stakeholders viewed consistency of messaging between the GP and the physiotherapist as vital to support deferral of imaging and optimise patient engagement with a self‐management approach *(Social support [unspecified] and information about others' approval)*.

Intervention components that required substantial organisational support or change were excluded due to the feasibility of future scale‐up. This included suggestions from stakeholders such as the provision of a specialist knee interface clinic for GPs, offering free access to physiotherapy within GP practices, and access to publicly funded personal trainers. Timely access to physiotherapy was identified as key to providing evidence‐based care; however, waiting times of 4–12 months within the public health service represented a substantial barrier. To overcome this, we negotiated with local physiotherapy management to enable access within 2–3 weeks to a 1‐h physiotherapy session (restructuring physical environment). Accessing this early session will promote GP engagement with the intervention and will not disqualify patients from accessing further physiotherapy outside this intervention, should they so choose.

### 3.4 The Final Intervention

The final selected BCTs, including content and mode of delivery, mapped to TDF domains and barriers are shown in Tables 2 and 3. Several additional elements, which did not map to specific barriers, were incorporated to enhance intervention implementation and GP engagement.

1. For training purposes, GP stakeholders specified a preference for workshops to be incentivised with Continuing Professional Development credits (*self‐incentive*).
2. Social influences are important in clinical practice, and interventions to change social norms can be effective in changing the clinical behaviours [104]**;** therefore, GPs who are influential amongst their peers (GP tutor × 1, clinical academic GP × 1 and GP leads of trainee scheme × 1) will be purposively invited to attend these small group workshops to leverage their social influence.

The final intervention was based on what was likely to be feasible, acceptable and relevant locally. It consisted of small group educational workshops targeted at GPs and the provision of GP educational resources to facilitate delivery of an ‘enhanced consultation’ to patients. GPs had the facility to refer patients to an early ‘best practice’ physiotherapy session. The patient‐targeted intervention had two main steps: an ‘enhanced consultation’ with the GP, followed by a ‘best practice’ physiotherapy session. An overview of the final intervention with rationale for inclusion of the intervention components based on APEASE criteria is presented in Table 4. The Patient Advice Leaflet was modified to incorporate feedback from stakeholders. This is mainly related to the accessibility of the language, and PwLE stakeholders also recommended highlighting the evidence proving arthroscopy was not more effective than sham surgery. GP stakeholders recommended that an electronic version of the resource should be made available.

**Table 4:** Summary of final intervention components with rationale for inclusion based on APEASE criteria.

	Intervention component and mode of delivery	Rationale for inclusion based on research team consideration of the *APEASE* * criteria
GP level	Interactive small group workshops (accredited with GP CPD points) delivered by experienced MSK physiotherapists. Workshops include: (i) update on evidence (use of exercise v's surgery, weight management, imaging indications, practice guidelines for DMT/early OA), (ii) conducting focused knee exam (red flags, differential diagnosis), (iii) communication tips and navigating discussion around need for imaging and (iv) delivering components of GP's ‘enhanced consultation’	Interactive workshops have demonstrated **effectiveness** (as part of multi‐component strategies); **acceptable** to GP stakeholders; **affordable** and **practicable** to deliver within the scope of the current project
GP level	Provision of GP resources (hard and digital copies) to support consultation including: (i) a quick reference guide to aid diagnosis, (ii) a quick reference guide on key aspects of management and ‘enhanced consultation’, (iii) a Patient Advice Leaflet and (iv) exercise prescription resources.	**Affordable** and **practicable** to deliver within the scope of the current project; **acceptable** to GP stakeholders
GP level	Self‐complete checklist on components of GP's ‘enhanced consultation’ embedded into physiotherapy referral form	Checklist **practicable** for GPs to complete as part of the physiotherapy referral process; **acceptable** to GP stakeholders
GP level	Written feedback to GP via physiotherapist discharge summary (re‐enforcing accuracy of GPs' clinical diagnosis and patient suitability for conservative management)	**Practicable** and **affordable** means of delivering feedback and reinforcing GP behaviour. Physiotherapist discharge summary is already part of routine care; **acceptable** to GP and physiotherapist stakeholders
GP level	Facility to refer patients to an early ‘Best Practice’ physiotherapy session (appointment within 2–3 weeks of GP consultation)	Demonstrates **equity** as all patients are entitled to access*; **acceptable** to all stakeholders
Patient level	GP ‘enhanced consultation’ to include: (i) clinical knee exam, (ii) verbally delivered educational messages, (iii) Patient Advice Leaflet and (iv) management plan agreed and documented on Patient Advice Leaflet	Individual components of consultation **acceptable** to GP and PwLE stakeholders; GP's ‘model consultations’ have previously demonstrated **effectiveness;** delivery mode deemed **practicable** as it fits within existing GP practice; no **side effects** or **safety** issues identified with delaying referral (restricting GPs' ability to refer for non‐indicated imaging may have presented safety issues)
Patient level	Patient Advice Leaflet designed to reassure and explain diagnosis, reinforce verbally delivered educational messages and help guide pain monitoring during activity. Section for co‐creating a treatment plan, simple starter exercises with progressions and QR codes linking to exercise videos	Component is **practicable** and **affordable; acceptable** to GP and PwLE stakeholders
Patient level	Physiotherapy ‘Best Practice’ session encompassing: (i) individualised patient education on likely prognosis, beliefs about pain, physical activity, weight management and other lifestyle factors; (ii) education on self‐management skills such as load management, activity modification and monitoring pain response; (iii) functional or physical activity goals identified and action plan created; (iv) introduction of logbook to self‐monitor physical activity and (v) graded exercise programme linked to functional goals, practised with physio, with options to progress and regress exercises	A similar single session approach has demonstrated **effectiveness** and is practicable to deliver; **acceptable** to GP, PwLE and physiotherapist stakeholders; no **side effects** or **safety** issues identified; demonstrates **equity** as all patients are entitled to access**

Abbreviations: CPD, continuous professional development; DMT, degenerative meniscal tear; MSK, musculoskeletal; OA, osteoarthritis; PwLE, People with Lived Experience of knee pain.

* *APEASE* criteria evaluating Acceptability, Practicability, Effectiveness, Affordability, Side effects (and safety) and Equity (74).

** Free physiotherapy through the Irish public health service is not universally available, and access is often restricted to holders of a medical card, which is means tested.

### 3.5 Step 4: How Can Behaviour Change Be Measured and Understood?

The intervention aims to facilitate healthcare professional and patient behaviour change. Behavioural analysis indicated that GPs require greater confidence, knowledge and skills to modify clinical practice. Training workshops will enhance self‐efficacy in musculoskeletal assessment, patient education and exercise prescription, while increasing motivation to adopt evidence‐based care and reduce unnecessary imaging and specialist referral. Communication training and practical strategies to manage patient expectations are expected to further reduce referrals, with the physical examination promoted as a means of reassurance and reframing pain, rather than solely a diagnostic task. Early access to physiotherapy will address organisational barriers to optimal care.

Patient behaviour change is expected through interactions with both GPs and physiotherapists. GPs will foster positive recovery expectations through an enhanced consultation and facilitate access to evidence‐based physiotherapy. Both professionals will work to modify beliefs about exercise and surgery and enhance self‐efficacy by providing support and guidance for self‐management and increased physical activity.

A logic model (Figure 2) of the GP and patient‐level interventions, mapped to hypothesised mechanisms of change and outcomes, was developed. We also outlined outcomes designed to capture change in GP and patient behaviour for measurement in a feasibility study (Table 5).

**Figure 2:**
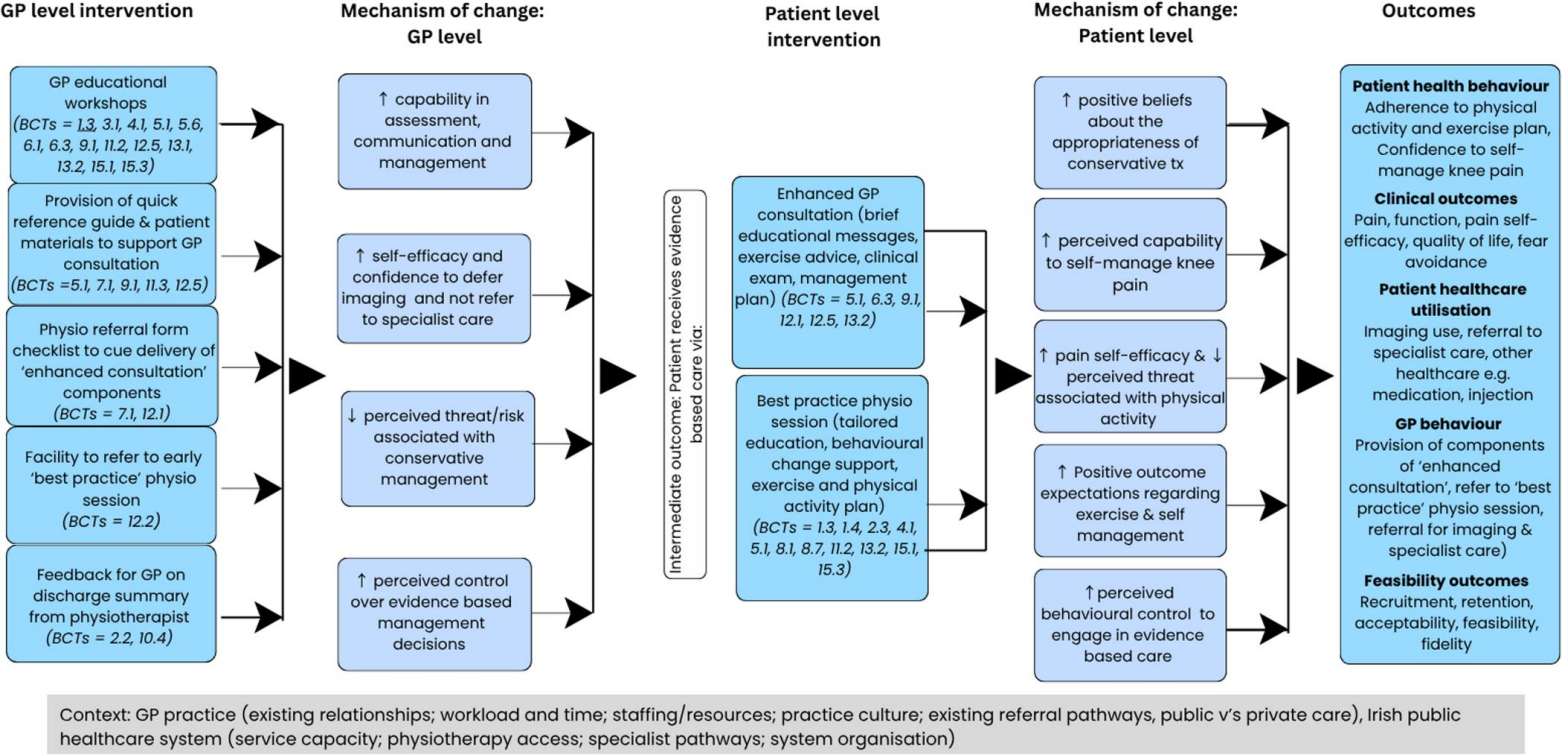
Theory‐informed logic model of the professional and patient‐level intervention mapped to hypothesised mechanisms of change and potential outcomes. BCT numbers are explained in the text of Tables 2 and 3. BCT = behaviour change technique, tx = treatment.

**Table 5:** Outcome measures to capture change in behaviour and clinical outcomes.

What to measure?		How to measure?
GP behaviours	GP performs clinical assessment (includes physical exam) and provides clinical diagnosis	Self‐report by GP using checklist on physiotherapy referral form Patient self‐report survey following GP consultation
	Imaging and secondary care referral rate	Record using a patient survey
	Patient referred to ‘best practice’ physiotherapy session	Audit of patient file
	Patient provided with educational messages and brief exercise advice	Self‐report by GP using checklist on physiotherapy referral form Patient self‐report survey following GP consultation
Patient behaviour	Engagement with the prescribed exercise and physical activity plan	Self‐rating (0–10) of adherence by patient survey Assessed using qualitative data from patient interviews
Patient health outcomes	Knee pain	Knee injury and Osteoarthritis Outcome Score (KOOS) Pain subscale
	Knee function	Knee Injury and Osteoarthritis Outcome Score (KOOS) Physical Function Short Form
	Pain self‐efficacy and fear avoidance beliefs	Short‐Form Pain Self‐Efficacy Scale (PSEQ‐2) and Fear Avoidance Beliefs Questionnaire (FABQ)
	Health‐related quality of life	5Q‐5D‐5L Quality of Life EuroQol 5 Dimension (EQ‐5D‐5L)
Feasibility outcomes	Recruitment and retention (GPs and patients)	Study log
	Intervention acceptability and feasibility	Assessed using qualitative data from patient and GP interviews Theoretical Framework of Acceptability Questionnaire Satisfaction with care is self‐rated on an ordinal scale

## 4 Discussion

### 4.1 Summary of Main Findings

This study describes the systematic development of a multi‐level, theory‐informed implementation intervention designed to improve evidence‐based management of DMT and early knee OA. By integrating behavioural theory, stakeholder input and empirical evidence, our intervention directly addresses well‐documented evidence–practice gaps in the conservative management of degenerative knee conditions.

#### 4.1.1 Comparison With Existing Literature

Through its explicit focus on early‐stage disease, this work addresses an important gap in the OA literature. To our knowledge, it is the first intervention designed to specifically target patients with DMT and early OA, a group often funnelled prematurely towards imaging and surgical consultation, having bypassed the core treatments of education and exercise. To date, much of the research involving patients with DMT has focused on comparing the effectiveness of non‐surgical approaches to arthroscopic surgery [29, 105, 106]. The current study takes the next logical step, which is to focus on implementing this evidence by designing an intervention to address the real‐world barriers to both the delivery and uptake of exercise and education for this patient cohort with early degenerative changes [36, 51]. A related implementation approach was taken in the UK National Health Service through the Personalised Knee Improvement Programme (P‐KIP), which was developed specifically to address behavioural and service‐level barriers to reducing arthroscopy for knee OA [107] and provide an evidence‐based conservative care pathway as an alternative. However, P‐KIP sought to substitute conservative care (education, physiotherapy and dietetic support) for arthroscopy, once patients with established knee OA had reached secondary care [108]. Meanwhile, the current intervention targets an earlier point in the care pathway by supporting evidence‐based management at the primary care consultation, before patients progress to imaging and specialist referral. Nonetheless, P‐KIP provides a useful real‐world example of de‐implementing arthroscopy, and its service evaluation illustrates the potential value of providing clinicians with a ‘substitute’ pathway to established low‐value care pathways. Our intervention adopts a similar approach and attempts to provide GPs with a credible alternative, via the provision of educational resources for patients and early access to a ‘best practice’ physiotherapy session, rather than focusing solely on reducing inappropriate clinical behaviours related to imaging and early referral to secondary care.

The behavioural analysis underpinning our intervention highlights that the evidence practice gaps are not driven by lack of knowledge alone, but by complex interactions between clinician beliefs and confidence, patient expectations, social norms, and system‐level constraints. These findings are consistent with prior work suggesting that implementing evidence‐based care for OA is a complex behavioural and organisational problem, not simply an information problem [45, 58, 69, 109]. A recently published evidence synthesis of barriers and enablers to the implementation of primary care‐based OA management programmes highlights the importance of the health system environment, with over one‐third of barriers and enablers relating to the broader health system [45]. Difficulty accessing care in the public sector, long delays, workforce shortages and patients being prematurely waitlisted are challenges to the provision of high‐quality OA care, both in Ireland and internationally [58, 110]. Health system capacity and resources were also cited as prominent barriers to evidence‐based care for early OA and DMTs in our study. This implies that implementation cannot rely solely on changing GP and patient behaviour, and the feasibility of scaling up this intervention will also depend on sufficient physiotherapy capacity to provide timely access to evidence‐based care. In Ireland, there is a critical need to build workforce capacity and ensure adequate resourcing of primary care physiotherapy [111]. Addressing these health system and service barriers requires health system and policy reform, including financing models that better support and incentivise high‐value OA care [110]. Future development work should also include policy‐makers to prioritise the implementation of current OA models of care into practice.

Another important barrier identified in our needs analysis, but considered broadly beyond the scope of the current study, was societal narratives and unhelpful beliefs about a predominantly structural model of musculoskeletal pain. Patient beliefs about the need for surgery and imaging were reinforced by their wider social circle. Such beliefs are recognised to contribute to low‐value care by shaping people's expectations of care and how they interact with clinicians [112, 113]. Comprehensively addressing these population‐level beliefs and promoting a narrative that supports active self‐management and physical activity necessitates public health campaigns to provide a broader re‐framing of musculoskeletal pain in society [114].

#### 4.1.2 Strengths and Limitations

A major strength of this study is the extensive stakeholder engagement across PwLE, GPs, physiotherapists and orthopaedic surgeons. We specifically chose the four‐step design approach by French et al. as it allows for the integration of stakeholder perspectives [59]. Stakeholder involvement is increasingly recognised as essential for enhancing intervention acceptability and feasibility, particularly for complex interventions implemented in real‐world healthcare settings [67, 68]. Stakeholder feedback meaningfully shaped target behaviours, intervention components and modes of delivery, thereby increasing the likelihood of successful implementation. For the purposes of conducting this stakeholder engagement, we chose to meet with groups of stakeholders separately. This decision was driven by evidence suggesting this approach is more acceptable and useful to stakeholders, who can feel their opinion is undervalued in a mixed group of HCPs and patients, compared to combining multiple stakeholders in a single group [115]. Nonetheless, this approach is more time‐consuming, involving multiple meetings, six in this study, with additional stakeholder engagement via email. The use of the widely used TDF framework to identify modifiable behavioural determinants strengthens the rigour and transparency of the intervention design process. Explicitly naming the hypothesised mechanisms of change means they can be specifically tested in future investigations.

Several limitations should be acknowledged. Firstly, qualitative data underpinning the behavioural analysis and stakeholder input were drawn from a single healthcare region. Specific local contextual factors may limit transferability to other healthcare settings with different funding, infrastructure and access. Furthermore, the use of existing qualitative datasets, while focused on the target cohort (people with DMT or early OA), meant the analysis was limited to issues explored within the original interviews. Secondly, while broad stakeholder perspectives were sought, a formal consensus methodology such as a Delphi process was not employed. Thirdly, organisational barriers such as long physiotherapy waiting lists were mitigated pragmatically through discussions with local health service management, rather than addressing this problem at a systems level, which may affect the potential to scale up the intervention. Fourthly, this intervention focused on education and exercise. Weight management is another key aspect of knee OA care. While this was covered in the GP education workshop, we did not have the resources to facilitate access to individualised weight management support. This aspect of care is arguably even more important for individuals with early OA, given that future weight gain and adiposity drive disease progression [116]. Finally, empirical evidence to prove theory‐driven interventions demonstrate greater effectiveness in changing professional behaviour is still sparse [117], as is the evidence to support their effectiveness for superior clinical outcomes [51]; therefore, testing of this intervention in practice is key. Until this, its acceptability, fidelity and effectiveness remain uncertain.

#### 4.1.3 Implications for Policy, Practice and Future Research

In terms of clinical implications, if this intervention is feasible and effective, it has the potential to reduce low‐value imaging and surgical referral, promote earlier engagement with self‐management, and improve patient confidence in conservative care. Embedding early consistent, evidence‐based messages at the patient–practitioner interface may also reduce fear‐driven healthcare utilisation and support more sustainable models of musculoskeletal care. Given the growing burden of OA disease and its associated comorbidities, this intervention aligns with a new focus on secondary prevention of early knee OA [118]. This intervention provides a structured example of intervening early, providing patients with a tailored plan and potentially influencing the subsequent disease process.

The next stage of this project will be a feasibility study to help address outstanding uncertainties in relation to the intervention. It is important to understand if the ‘enhanced GP consultation’ and the ‘best practice’ physiotherapy session are acceptable to patients and HCPs and feasible to deliver within the Irish public healthcare system. A mixed‐methods approach will be used, including qualitative interviews with patients and GPs to explore experiences of the intervention, perceived usefulness, and barriers and facilitators to participation and delivery. Acceptability will be assessed using the Theoretical Framework of Acceptability (TFA) questionnaire [119], while satisfaction with care will be measured using a self‐rated ordinal scale. Potential behavioural outcomes include changes in GP management practices, such as imaging and secondary care referrals and patient engagement with recommended self‐management strategies. Feasibility will additionally be assessed through recruitment and retention rates for GPs and patients, providing an indication of the ability to engage and retain patients attending primary care as study participants. Findings will inform further refinement of the intervention and the feasibility of progressing to a full trial. Finally, a feasibility study will also provide an opportunity to further test and refine educational materials for patients and HCPs. For example, ensuring the accessibility of the Patient Advice Leaflet is an important consideration, including a formal assessment of readability, colour contrast and digital accessibility, or potentially developing alternative formats such as large‐print, audio or Easy Read versions to ensure equitable access across diverse patient groups.

## 5 Conclusion

Using a systematic, theory‐informed and stakeholder‐engaged approach, we developed a multifaceted intervention to support delivery and uptake of evidence‐based care for DMT and early knee OA. The intervention comprised small‐group educational workshops and resources for GPs to support the delivery of an ‘enhanced consultation’, with the facility to refer patients for an early ‘best practice’ physiotherapy session. The patient intervention, therefore, consisted of two key components: an ‘enhanced consultation’ with the GP followed by a ‘best practice’ physiotherapy session. This single session delivers individualised education and a bespoke exercise and physical activity plan to boost self‐management and self‐efficacy. The resulting intervention requires testing to explore its acceptability, feasibility and suitability for scale‐up in clinical practice.

## Author Contributions

**Helen O'Leary:** conceptualisation, investigation, funding acquisition, writing – original draft, methodology, formal analysis. **Elaine Toomey:** methodology, writing – review and editing. **Helen P. French:** methodology, funding acquisition, writing – review and editing. **Liam Glynn:** methodology, writing – review and editing, funding acquisition. **Katie Robinson:** funding acquisition, methodology, writing – review and editing. **Liam G. Ryan:** funding acquisition, methodology, writing – review and editing. **Clodagh Toomey:** funding acquisition, writing – review and editing, methodology. **Hazel Jenkins:** methodology, writing – review and editing. **Karen McCreesh:** conceptualisation, investigation, funding acquisition, writing – review and editing, supervision, formal analysis.

## Ethics Statement

All recruitment and data collection procedures received approval from the University of Limerick Faculty of Education and Health Science Research Ethics Committee (Project ID: 2023_02_20_EHS).

## Consent

Consent to participate was obtained from all participants.

## Conflicts of Interest

The authors declare no conflicts of interest.

## Supporting information


Supplementary File 1

Supplementary File 2

## Data Availability

The data for this study will not be shared, as we do not have permission from the participants or ethics approval to do so.
