# Supplementary material for: Supporting the Uptake of Evidence‐Based Care for Degenerative Meniscal Tears and Early Knee Osteoarthritis: A Collaborative Approach to the Development of an Intervention Using Stakeholder Perspectives and Integrating Behaviour Change Theory
Source: Health Expect. 2026 Nov 6;29(6):e70940. doi: 10.1111/hex.70940 (PMC13742377; doi:10.1111/hex.70940)
Supplement: Supplementary file 1 [file HEX-29-e70940-s001.docx]

| **Supplementary File 2.**  Table A. Initial list of potential BCTs mapped to GP target behaviours and TDF intervention functions  Table B. Initial list of potential BCTs mapped to patient target behaviour and TDF function  Table C. Rationale for excluding BCTs at Step 3 to address GP target behaviours  Table D. Rationale for excluding BCTs at Step 3 to address patient target behaviour | | | |
| --- | --- | --- | --- |
| **Table A. Initial list of potential BCTs mapped to GP target behaviours and TDF intervention functions** | | | |
| **Target behaviour 1. GP provides patient with diagnosis based on clinical history and physical examination** | | | |
| **Barriers** | **TDF Domain** | **Initial list of considered BCTs (final selection in bold font)** | |
| Time pressures: Limited consultation time reduces likelihood of MSK exam and increases reliance on MRI | Environmental context and resources | **Conserving mental resources**  **Adding objects to the environment**  Restructuring physical environment  Restructuring the Social Environment  Prompts/cues  Avoidance/reducing exposure to cues for the behaviour  Removing aversive stimulus  Social support (practical) | |
|  |  | **Time management*** | |
| Low confidence in MSK skills: Uncertainty about MSK exam | Belief about capabilities | **Demonstration of the behaviour**  **Instruction on how to perform the behaviour**  **Verbal persuasion about capability**  **Focus on past success**  Self-talk  Problem solving  Mental rehearsal of successful performance  Graded tasks  Behavioural practice/rehearsal | |
|  | Skills | Behavioural practice/rehearsal  **Instruction on how to perform the behaviour**  Graded tasks | |
| Knee pain is not a priority: Consultation focus on other comorbidities | Memory, attention and decision-making processes | **Conserving mental resources**  **Prompts/cues**  Restructuring the physical environment*  Action planning* | |
| Fear of misdiagnosis and litigation: Anxiety about missing serious pathology | Belief about consequences | **Information about health consequences**  Pros and Cons  **Information about emotional consequences**  Reward (outcome)  Material incentive  Salience of consequences  Information about social and environmental consequences  Anticipated regret  Incentive (outcome)  Comparative imagining of future outcomes | |
| **Target Behaviour 2: GP defers or avoids non-recommended care (imaging and early referral to specialist care) at least until patient has accessed physiotherapy** | | | |
| **Barriers** | **TDF Domain** | **Potential BCT** | |
| Patient expectations and fears of damaging therapeutic relationship: GP concede to patient MRI request | Social influences | **Information about others’ approval**  Social support (unspecified)  Social comparison  Social support (practical)  Social reward | |
|  | Emotion | **Reduce negative emotions** | |
|  |  | **Demonstration of the behaviour*** | |
| Desire for a specialist opinion to direct management; uncertain about the indications for surgery or exercise | Belief about capabilities | **Instruction on how to perform the behaviour**  **Demonstration of behaviour**  Verbal persuasion about capability  Problem solving  Focus on past success  Self-talk  Graded tasks  Behavioural practice or rehearsal  **Social reward** | |
|  | Knowledge | **Information about health consequences**  **Instruction on how to perform the behaviour**  Information about social and environmental consequences  Information about antecedents  Biofeedback | |
| Long wait to access secondary care and perceived lack of other management options | Environmental context and resources | **Restructuring physical environment**  **Adding objects to the environment**  Restructuring the social Environment  Prompts/cues  Avoidance/reducing exposure to cues for the behaviour  Remove aversive stimulus  Social support (practical) | |
| Awareness of the potentially negative consequences of imaging (enabler) | Beliefs about consequences | **Information about health consequences**  Pros and Cons  Information about emotional consequences  Reward (outcome)  Material incentive  Salience of consequences  Information about social and environmental consequences  Anticipated regret  Incentive (outcome)  Comparative imagining of future outcomes | |
| Confidence in ability to leverage existing trusting relationship with patient to delay non-recommended care (enabler) | Beliefs about capabilities | Behavioural practice or rehearsal  Information about health consequences  Instruction on how to perform the behaviour  Demonstration of behaviour  Verbal persuasion about capability  Problem solving  **Focus on past success**  Self-talk  Mental rehearsal of successful performance  Graded tasks | |
| **Target Behaviour 3. GP provides patient with the recommended first line treatment of education and exercise** | | | |
| **Barriers** | **TDF Domain** | **Potential BCTs** |  |
| Patients perceived by GP to be disinterested in exercise and reluctant to engage with exercise-based care | Social influences | **Social reward**  **Information about other’s approval**  **Social comparison**  Social support (practical)  Social support (unspecified) | |
| Lack of GP skills, and confidence in providing education and exercise | Beliefs about capabilities | Graded tasks  Behavioural practice/rehearsal  **Instruction on how to perform the behaviour**  **Demonstration of behaviour**  **Verbal persuasion about capability**  Problem solving  Focus on past success  Self-talk | |
|  | Skills | Behavioural practice/rehearsal  **Instruction on how to perform the behaviour**  Graded tasks | |
| Exercise prescription and education is perceived as the physiotherapist’s domain | Professional role and identify | **Identify associated with changed behaviour**  **Credible source**  Social comparison  **Social support** | |
| Lack of resources to support delivery of patient education and exercise prescription by GP | Environmental context and resources | Restructuring the physical environment  Restructuring the social environment  Social support (practical)  Prompts/cues  Remove aversive stimuli  **Adding objects to the environment**  Avoidance/reducing exposure to cues for the behaviour | |
| Limited access to publicly funded physiotherapy services and prohibitive cost of private physiotherapy | Environmental context and resources | **Restructuring the physical environment**  Restructuring the social environment  Social support (practical)  Prompts/cues  Remove aversive stimuli  Adding objects to the environment  Avoidance/reducing exposure to cues for the behaviour | |
| GPs value physical activity as part of management (enabler) | Beliefs about consequences | **Information about health consequences**  Pros and Cons  Information about emotional consequences  Framing/Reframing  Reward (outcome)  Material incentive  Salience of consequences  Information about social and environmental consequences  Anticipated regret | |
| Feelings of frustration and guilt with available treatment options (enabler) | Emotion | Reduce negative emotions  **Info about emotional consequences**  Framing/reframing  Anticipated regret | |
| BCT, behaviourak change technique; MSK, musculoskeletal; TDF, Theoretical Domains Framework.  * included in initial list in response to stakeholder suggestions | | | |

| **Table B. Initial list of potential BCTs mapped to patient target behaviour and TDF function** | | |
| --- | --- | --- |
| **Patient target behaviour. Patient engages with non-surgical management for their knee pain following consultation with the GP and physiotherapist** | | |
| **Barriers** | **TDF Domain** | **Initial list of considered BCTs potential BCTs (final selection in bold font)** |
| Negative expectations of conservative management: Surgery perceived as necessary | Beliefs about consequences | **Information about health consequences**  Pros and Cons  Information about emotional consequences  **Framing/Reframing**  Reward (outcome)  Material incentive  Salience of consequences  Information about social and environmental consequences  Anticipated regret |
| Poor engagement and adherence with exercise/physical activity: Rehabilitation perceived as a more difficult treatment option | Intentions; | **Goal setting**  **Information about health consequences**  Incentive (outcome) |
|  | Behavioural regulation | **Problem solving**  **Self-monitoring of behaviour**  Information about antecedents  Behaviour substitution  Reduce negative emotions  Conserving mental resources |
|  |  | **Action planning*** |
| Ability to exercise (or be physically active) hampered by lack of knowledge & confidence | Belief about capabilities | **Graded tasks**  **Behavioural practice/rehearsal**  **Instruction on how to perform the behaviour**  Demonstration of behaviour  **Verbal persuasion about capability**  Problem solving  **Focus on past success**  Self-talk |
|  | Knowledge | Information about health consequences  **Instruction on how to perform the behaviour**  Information about social and environmental consequences  Information about antecedents  Biofeedback |
| Fearful of doing harm with exercise | Emotion | **Reduce negative emotions**  Info about emotional consequences  **Framing/reframing**  Anticipated regret |
| Inadequate access to physiotherapy or access to non-evidence-based physiotherapy | Environmental context and resources | **Restructuring the physical environment**  Restructuring the social environment  Social support (practical)  Prompts/cues  Remove aversive stimuli  **Adding objects to the environment**  Avoidance/reducing exposure to cues for the behaviour |
| Perception that a structural diagnosis (using MRI) is essential before treatment | Beliefs about Consequences | **Information about health consequences**  Pros and Cons  Information about emotional consequences  **Framing/Reframing**  Reward (outcome)  Material incentive  Salience of consequences  Information about social and environmental consequences  Anticipated regret |
|  |  | Credible source* |
| Reinforcement of MRI/surgery beliefs by others | Social influences | Social reward  **Information about other’s approval**  Social comparison  Social support (practical)  Social support (unspecified) |
| Positive past experiences with exercise (enabler) | Reinforcement | Material incentive (behaviour)  Material reward (behaviour)  **Non-specific reward**  Social reward  Non-specific incentive  Incentive (outcome)  Punishment  Reward (outcome) |
| Strong desire to return to valued activities (enabler) | Motivation | **Goal setting**  Feedback on behaviour  Pros and cons  **Incentive (outcome)**  Reward (outcome)  Self talk |
| BCT, behavioural change technique; MRI, magnetic resonance imaging; TDF, Theoretical Domains Framework. * BCT included in response to stakeholder feedback. | | |

| **Table C. Rationale for excluding BCTs at Step 3 to address GP target behaviours** | | | |
| --- | --- | --- | --- |
| **Target behaviour 1. GP provides patient with diagnosis based on clinical history and physical examination** | | | |
| **Barriers** | **TDF Domain** | **Proposed BCT** | **Rationale/decision making for excluding key BCTs suggested by the Theory and Techniques Tool** |
| Time pressures: Limited consultation time reduces likelihood of MSK exam and increases reliance on MRI | Environmental context and resources | Restructuring the physical environment | Could integrate new knee assessment template or prompts into GP electronic medical record **BUT** deemed not feasible by GP stakeholders |
|  |  | Prompts and cues | Overlap with selected BCTs (conserving mental resources/adding objects to the enviroment) |
|  |  | Remove aversive stimulus  Avoidance/reducing exposure to cues for the behaviour | Not feasible to remove exposure to time pressures |
|  |  | Social support (practical)  Restructuring the social enviroment | Deemed not relevant to addressing this specific barrier by researchers |
| Low confidence in MSK skills: Uncertainty about MSK exam | Belief about capabilities; Skills | Mental rehearsal of successful performance  Self-talk | Could ask GPs to imagine themselves successfully completing the behaviour or use positive self-talk **BUT** deemed unsuitable as likely to translate to GP consultation |
|  |  | Graded tasks  Problem solving | Could use to build GP mastery of MSK skills **BUT** deemed unfeasible in GP consultation or GP workshop due to time pressures |
|  |  | Behavioural practice/rehearsal | Use workshops to facilitate GPs practicing examination skills **BUT** lack of time and equipment (plinths/exam tables) |
| Knee pain is not a priority: Consultation focus on other comorbidities | Memory, attention and decision-making processes | Restructuring the physical environment | Could integrate new reminders or prompts into electronic medical record **BUT** deemed unfeasible within project scope by researchers |
|  |  | Action planning | GPs could arrange a follow-up appointment focused solely on knee pain **BUT** Deemed not acceptable by some GP stakeholders due to high demand for appointments. |
| Fear of misdiagnosis and litigation: Anxiety about missing serious pathology | Belief about consequences | Pros and Cons  Salience of consequences  Anticipated regret | Could incorporate these BCTs by presenting and discussing case examples at GP workshop **BUT** imaging use is not the sole focus of the workshop and GP stakeholders expressed preference for emphasis on other aspects e.g. clinical exam , exercise prescription |
|  |  | Reward (outcome)  Material incentive  Incentive (outcome) | Could provide reward for not ordering imaging **BUT** researchers identified ethical issues and not acceptable to stakeholders |
|  |  | Information about social and environmental consequences | Deemed not relevant to address this specific barrier by researchers |
| **Target Behaviour 2: GP defers or avoids non-recommended care (imaging and early referral to specialist care) at least until patient has accessed physiotherapy** | | | |
| **Barriers** | **TDF Domain** | **Potential BCT** | **Rationale for excluding BCTs at Step 3** |
| Patient expectations and fears of damaging therapeutic relationship: GP concede to MRI request | Social influences; Emotion | Social support (unspecified) | Could encourage GPs to discuss challenging cases with peers **BUT** intervention not targeted at practice level so may not be feasible |
|  |  | Social comparison | Demonstrate how peers deliver guideline concordant care in similar situations **BUT** overlaps with other BCT info about other’s approval |
|  |  | Social support (practical) | Provide tools that make adherence easier e.g. generic explanations for not ordering MRI **BUT** GP stakeholders in time pressed consultation unlikely to use and express preference for individualised care |
|  |  | Social reward | Deemed not relevant to address this specific barrier by researchers |
| Desire for a specialist opinion to direct management; uncertain about the indications for surgery or exercise | Belief about capabilities; Knowledge | Self-talk | Researchers deemed unsuitable as likely to translate to GP consultation |
|  |  | Verbal persuasion about capability  Focus on past success | Overlap with selected BCT (social reward) Written feedback |
|  |  | Graded tasks | Deemed not relevant to address this specific barrier by researchers |
|  |  | Behavioural practice or rehearsal | Could have GPs reherse prescribing exercise **BUT** insufficient time in workshop |
|  |  | Problem solving | Could support GPs to identify barriers to deferring/avoiding secondary care referral and identifying **BUT** time consuming to practice in GP workshop |
|  |  | Information about social and environmental consequences | Could expands GP knowledge beyond health to wider impacts **BUT** deemed by researchers to have limited revalance to this specific barrier |
|  |  | Information about antecedents | Could provide GPs would likely precursors to onward referral **BUT** deemed by researchers unfeasible given complex and individualised nature of each case |
|  |  | Biofeedback | Deemed not relevant to addressing this specific barrier |
| Long wait to access secondary care and perceived lack of other management options | Environmental context and resources | Restructuring the Social Environment | Could alter referral pathways to secondary care orthoapedic clinics (making it mandatory to attend physiotherapy first) **BUT** not acceptable to GP and PWLE stakeholders and deemed not feasible within project scope |
|  |  | Prompts/cues | External reminders embedded in the environment **BUT** |
|  |  | Avoidance/reducing exposure to cues for the behaviour  Removing aversive stimulus  Social support (practical) | Deemed by researchers to have limited revalance to this specific barrier |
| **Target Behaviour 3. GP provides patient with the recommended first line treatment of education and exercise** | | | |
| **Barriers** | **TDF Domain** | **Potential BCTs** | **Rationale/decision making for excluding key BCTs suggested by the Theory and Techniques Tool** |
| GP perceive patients to be disinterested in exercise and reluctant to engage with exercise-based care | Social influences | **Social support (unspecified)** | Could facilitate peer discussion where GPs share experiences of patients successfully engaging with conservative management **BUT** could be time consuming to facilitate in workshop |
|  |  | Restructuring the social environment | Could target culture at GP practice level and embed routine exercise prescription into culture of practice **BUT** not feasible to influence practice level behaviours unless all GPs attend |
|  |  | Social comparison | Could use other GPs’ behaviours as a benchmark **BUT** data on exercise referral rates not readily available and deemed unfeasible by researchers |
|  |  | Social reward | Overlap with other selected BCTs e.g. Info on Other Approval |
| Lack of GP skills, and confidence in providing education and exercise | Beliefs about capabilities; Skills | Graded tasks | Could use to build GP mastery of exercise prescription **BUT** deemed unfeasible in once-off workshop by researchers |
|  |  | Self-talk | Deemed unsuitable as unlikely to translate easily to GP consultation |
|  |  | Problem solving | Could support GPs to identify barriers to exercise **BUT** time consuming to practice in GP workshop |
|  |  | Feedback on behaviour | Repeated performance to develop competence **BUT** insufficient time within GP workshop to facilitate this practice/rehearsal of exercise prescription |
|  |  | Focus on past success | Overlap with other selected BCT (Verbal persuasion about capabilities) |
| Exercise prescription and education is perceived as the physiotherapist’s domain | Professional role and identity | Social comparison | Could help GPs reinterpret this behaviour so it aligns with professional identity **BUT** overlap with other BCT with stronger link (identify associated with changed behaviour) |
| Lack of resources to support delivery of patient education and exercise prescription by GP | Environmental context and resources | Restructuring the physical environment  Restructuring the social environment  Social support (practical)  Prompts/cues  Remove aversive stimuli  Avoidance/reducing exposure to cues for the behaviour | Specific barrier best addressed by BCT ‘Adding objects to the environment’ based on GP consultation |
| Limited access to publicly funded physiotherapy services and prohibitive cost of private physiotherapy | Environmental context and resources | Adding objects to the environment  Social support (practical)  Prompts/cues  Remove aversive stimuli  Avoidance/reducing exposure to cues for the behaviour | Deemed inadequate to address this specific barrier by stakeholders and researchers |
|  |  | Restructuring the social environment | Could create new referral pathway to community exercise programme **BUT** considered outside the scope of the study |
| GPs value physical activity as part of management (enabler) | Beliefs about consequences | Pros and Cons  Information about emotional consequences  Framing/Reframing  Reward (outcome)  Material incentive  Salience of consequences  Information about social and environmental consequences  Anticipated regret | Selected BCT (Info about health consequences) deemed most appropriate to reinforce this enabler |
| Feelings of frustration and guilt with available treatment options (enabler) | Emotion | Anticipated regret | Overlap with selected BCT (Info about emotional consequences) |
| BCT, behavioural change technique; MSK, musculoskeletal; TDF, Theoretical Domains Framework | | | |

| **Table D. Rationale for excluding BCTs at Step 3 to address patient target behaviour** | | | |
| --- | --- | --- | --- |
| **Patient target behaviour (engages with non-surgical management for their knee pain following consultation with the GP and physiotherapist)** | | | |
| **Barriers** | **TDF Domain** | **Potential BCTs** | **Rationale for excluding BCTs** |
| Negative expectations of conservative management: Surgery perceived as necessary | Beliefs about consequences | Salience of consequences  Reward (outcome) | Could highlight meaningful benefits of staying active (pain reduction, improved function) **BUT** covered by other BCT (information about health consequences) |
|  |  | Pros and cons  Anticipated regret | Could use video testimonials to high-light Cons and discourage surgery **BUT** Stakeholders (especially PWLE) judged unacceptable; felt patient stories risked being perceived as coercive |
|  |  | Incompatible beliefs | Potential approach for addressing patient beliefs about surgery **BUT** covered adequately by other BCTs (Information about Health Consequences, Credible source) |
|  |  | Information about emotional consequences  Information about social and environmental consequences  Material incentive | Deemed not relevant to specific barrier |
| Poor engagement and adherence with exercise/physical activity: Rehabilitation perceived as a more difficult treatment option | Intentions; Behavioural regulation | Conserving mental resources | Simple reminders (e.g., logbook, phone reminders) **BUT** overlap with self-monitoring of behaviour |
|  |  | Reduce negative emotions | Encourages patient articulation of negative emotions **BUT** will be part of identifying barriers during physical activity goal setting and action planning process with physiotherapist |
|  |  | Problem solving | Support patient to identify barriers to rehab **BUT** overlap with other BCTs (action planning, goal setting) |
|  |  | Information about antecedents  Incentive (outcome) | Overlap with identification of facilitators to physical activity during goal setting  Overlap with goal setting |
| Ability to exercise (or be physically active) hampered by lack of knowledge & confidence | Belief about capabilities, Knowledge | Self-talk | Positive self-talk increases confidence while performing exercises **BUT** identified as too psychologically oriented; physiotherapists stakeholders felt it was beyond scope of a single-session approach |
|  |  | Feedback on behaviour | Immediate reinforcement from the physiotherapist **BUT** already encompassed as part of behavioural practice and rehearsal |
|  |  | Behavioural experiments | Behavioural experiments **BUT** not insufficient time within single physio session to perform |
|  |  | Problem solving  Information about antecedents | Overlap with goal setting physical activity and action planning |
|  |  | Demonstration of behaviour | Covered by other BCTs (Instruction on how to perform the behaviour, Behavioural practice/rehearsal) |
|  |  | Information about health consequences  Information about social and environmental consequences | Positive benefits of exercise addressed when targeting other barriers |
|  |  | Biofeedback | Deemed not relevant to identifying this specific barrier |
| Fearful of doing harm with exercise | Emotion | Info about emotional consequences | Overlap with selected BCT ‘Reduce negative emotions’ |
|  |  | Anticipated regret | Not considered motivating by patient stakeholders |
| Inadequate access to physiotherapy or access to non-evidence-based physiotherapy | Environmental context and resources | Restructuring the social environment  Social support (practical) | Could modify the social context or provide social support **BUT** deemed by researchers unlikely to adequately address this barrier |
|  |  | Problem-solving  Remove aversive stimuli  Avoidance/reducing exposure to cues for the behaviour  Prompts and cues | These researchers deemed by researchers unlikely to adequately address this barrier |
| Perception that a structural diagnosis (using MRI) is essential before treatment | Beliefs about Consequences | Information about health consequences | Covered adequately by other BCTs e.g. credible source, information about other’s approval |
|  |  | Salience of consequences  Anticipated regret  Pros and cons | Overlap with other selected BCTs that are more feasible to incorporate (Information about health consequences, Credible source) |
|  |  | Information about social and environmental  Information about emotional consequences  Reward (outcome)  Material incentive | Deemed not relevant by researchers |
| Reinforcement of MRI/surgery beliefs by others | Social influences | Social support (practical)  Social support (unspecified) | Social support (practical)- delivery of physio intervention in group setting would leverage social support **BUT** not practical to provide physio within 2 to 3 weeks of GP if want to deliver in group setting |
|  |  | Social comparison | Could present testimonials from other patients BUT PWLE stakeholders have preference for ‘expert’ opinion |
|  |  | Social reward | Deemed by researchers not relevant to addressing this specific barrier |
| Positive past experiences with exercise (enabler) | Reinforcement | Material incentive (behaviour)  Material reward (behaviour) | Deemed by researchers not relevant to addressing this specific barrier |
|  |  | Social reward  Non-specific incentive  Non-specific reward  Punishment  Reward (outcome) | Selected BCT (incentive outcome) deemed most appropriate to enhance this specific enabler |
| Strong desire to return to valued activities (enabler) | Motivation | Feedback on behaviour  Pros and cons | Aspects of these BCTs incorporated into selected BCT (goal setting) |
|  |  | Incentive (outcome)  Reward (outcome | Overlap with goal setting BCT, deemed more appropriate here |
|  |  | Self-talk |  |
| BCT, behavioural change technique; MRI, magnetic resonance imaging; TDF, Theoretical Domains Framework | | | |
