# Supplementary material for: Supporting the Uptake of Evidence‐Based Care for Degenerative Meniscal Tears and Early Knee Osteoarthritis: A Collaborative Approach to the Development of an Intervention Using Stakeholder Perspectives and Integrating Behaviour Change Theory
Source: Health Expect. 2026 Nov 6;29(6):e70940. doi: 10.1111/hex.70940 (PMC13742377; doi:10.1111/hex.70940)
Supplement: Supplementary file 2 [file HEX-29-e70940-s002.docx]

GUIDED – a guideline for reporting for intervention development studies.

|  | Item description | Explanation | Page in manuscript where item is located | Other* |
| --- | --- | --- | --- | --- |
| 1. | Report the context for which the  intervention was  developed. | Understanding the context in which an intervention was developed informs readers about the suitability and transferability of the intervention to the  context in which they are considering evaluating, adapting or using the intervention. Context here can include place, organisational and wider socio-political factors that may influence the development and/or delivery of the  intervention (15). | Pg 4 (Methods) |  |
| 2. | Report the purpose of the intervention  development process. | Clearly describing the purpose of the intervention specifies what it sets out to achieve. The purpose may be informed by research priorities, for example those identified in systematic reviews, evidence gaps set out in practice guidance such as The National Institute for Health and Care Excellence or specific prioritisation exercises such as those undertaken with patients and practitioners through the James Lind Alliance. | Pg 4 (Background) |  |
| 3. | Report the target  population for the  intervention  development process. | The target population is the population that will potentially benefit from the intervention – this may include patients, clinicians, and/or members of the public. If the target population is clearly described then readers will be able to understand the relevance of the intervention to their own research or practice. Health inequalities, gender and ethnicity are features of the target population that may be relevant to intervention development processes. | Pg 3 (Background) & Table 1 (Results) |  |
| 4. | Report how any  published  intervention  development  approach contributed  to the development  process | Many formal intervention development approaches exist and are used to guide the intervention development process (e.g. 6Squid (16) or The Person Based Approach to Intervention Development (17)). Where a formal intervention development approach is used, it is helpful to describe the process that was followed, including any deviations. More general approaches to intervention development also exist and have been categorised as follows (3):- Target Population-centred intervention development; evidence and theory-based intervention development; partnership intervention  development; implementation-based intervention development; efficacy- based intervention development; step or phased-based intervention development; and intervention-specific intervention development (3). These approaches do not always have specific guidance that describe their use. Nevertheless, it is helpful to give a rich description of how any published approach was operationalised. | Pg 4 (Methods) |  |
| 5. | Report how evidence  from different sources informed the intervention  development process. | Intervention development is often based on published evidence and/or primary data that has been collected to inform the intervention development process. It is useful to describe and reference all forms of evidence and data  that have informed the development of the intervention because evidence bases can change rapidly, and to explain the manner in which the evidence and/or data was used. Understanding what evidence was and was not available at the time of intervention development can help readers to assess transferability to their current situation. | Pg 10 (Results) |  |
| 6. | Report how/if  published theory  informed the  intervention  development process. | Reporting whether and how theory informed the intervention development process aids the reader’s understanding of the theoretical rationale that underpins the intervention. Though not mentioned in the e-Delphi or consensus meeting, it became increasingly apparent through the development of our guidance that this theory item could relate to either existing published theory or programme theory | Pg 6 (Methods) & Pg 8, Pg 9 (Methods) |  |
| 7. | Report any use of  components from an  existing intervention  in the current  intervention  development process. | Some interventions are developed with components that have been adopted from existing interventions. Clearly identifying components that have been adopted or adapted and acknowledging their original source helps the reader to understand and distinguish between the novel and adopted components of the new intervention. |  | The single session physiotherapy intervention component was adopted from a ‘best practice advice’ session developed for the Getting it Right: Addressing Shoulder Pain (GRASP) trial- *Keene, D.J., et al. (2020).*  [10.1016/j.physio.2019.07.002](https://doi.org/10.1016/j.physio.2019.07.002) |
| 8. | Report any guiding  principles, people or  factors that were  prioritised when  making decisions  during the intervention  development process | Reporting any guiding principles that governed the development of the application helps the reader to understand the authors’ reasoning behind the decisions that were made. These could include the examples of particular populations who views are being considered when designing the intervention,  the modality that is viewed as being most appropriate, design features considered important for the target population, or the potential for the intervention to be scaled up. | Pg 9, 10 (Step 3 Methods) |  |
| 9. | Report how  stakeholders  contributed to the  intervention  development process. | Potential stakeholders can include patient and community representatives, local and national policy makers, health care providers and those paying for or commissioning health care. Each of these groups may influence the intervention development process in different ways. Specifying how differing groups of stakeholders contributed to the intervention development process helps the reader to understand how stakeholders were involved and the degree of influence they had on the overall process. Further detail on how to integrate stakeholder contributions within intervention reporting are available (19). | Pg 5, 6, 8, 9 (Methods) & pg 10, 13, 14, 15 (Results) |  |
| 10. | Report how the  intervention changed in  content and format  from the start of the  intervention  development process. | Intervention development is frequently an iterative process. The conclusion of the initial phase of intervention development does not necessarily mean that all uncertainties have been addressed. It is helpful to list remaining uncertainties such as the intervention intensity, mode of delivery, materials,  procedures, or type of location that the intervention is most suitable for. This can guide other researchers to potential future areas of research and practitioners about uncertainties relevant to their healthcare context. | Pg 12, 13, 14, 15 (Results) |  |
| 11. | Report any changes to  interventions  required or likely to  be required for  subgroups. | Specifying any changes that the intervention development team perceive are  required for the intervention to be delivered or tailored to specific sub groups  enables readers to understand the applicability of the intervention to their  target population or context. These changes could include changes to  personnel delivering the intervention, to the content of the intervention, or to  the mode of delivery of the intervention | NA |  |
| 12. | Report important  uncertainties at the  end of the  intervention  development process. | Intervention development is frequently an iterative process. The conclusion of the initial phase of intervention development does not necessarily mean that all uncertainties have been addressed. It is helpful to list remaining uncertainties such as the intervention intensity, mode of delivery, materials, procedures, or type of location that the intervention is most suitable for. This can guide other researchers to potential future areas of research and practitioners about uncertainties relevant to their healthcare context | Pg 17 Discussion |  |
| 13. | Follow TIDieR  guidance when  describing the  developed  intervention | Interventions have been poorly reported for a number of years. In response to this, internationally recognized guidance has been published to support the high quality reporting of health care. Interventions and public health interventions. This guidance should therefore be followed when describing a developed intervention |  | Intervention reported following TIDieR guidance in our published feasibility study protocol ^63^ |
| 14. | Report the  intervention  development process  in an open access  format | Unless reports of intervention development are available people considering using an intervention cannot understand the process that was undertaken and make a judgement about its appropriateness to their context. It also limits cumulative learning about intervention development methodology and  observed consequences at later evaluation, translation and implementation stages. Reporting intervention development in an open access (Gold or Green) publishing format increases the accessibility and visibility of intervention development research and makes it more likely to be read and used. Potential platforms for open access publication of intervention development include  open access journal publications, freely accessible funder reports or a study web-page that details the intervention development process | NA |  |
|  |  |  |  |  |

- If item is reported elsewhere, then the location of the information can be stated here.

GRIPP 2 Short Form Version

| **Section and topic** | **Item** |
| --- | --- |
| **1: Aim**  Report the aim of PPI in the study | To ensure that every stage of the intervention development process was informed by their lived experiences, priorities, and practical needs of people with degenerative meniscal tears (DMT) and early knee osteoarthritis (OA) |
| **2: Methods**  Provide a clear description of the methods used for PPI in the study | For people with lived experience of knee pain (PwLE):   - Three in‑person meetings at early, middle, and late stages of intervention development were facilitated - Meetings involved structured discussions that fed into the 4-step intervention development process, facilitated by a researcher and a physiotherapist - PwLE were asked for feedback on all components of the draft intervention and patients resources   For clinicians:   - One online group meeting with GPs (n=6) - One in‑person meeting with physiotherapists (n=6) - Individual discussions with orthopaedic surgeons (n=2) and a health service manager - Follow‑up email consultations to refine intervention materials   All meetings were audio-recorded and analysed using content analysis |
| **3. Results**  Outcomes—Report the results of PPI in the study, including both positive and negative outcomes | PPI contributed to the study results in several ways, including:   - Refinement of target behaviours - Generating ideas for transforming BCTs into intervention content - Providing perspectives to inform decisions on which BCTs were selected or excluded - Identifying key patient information needs and shaping the educational messages delivered by clinicians and the Patient Advice Leaflet - Making suggestions to improve the comprehension of the patient materials - Helping populate the APEASE criteria (acceptable and practicable criteria) |
| **4. Discussion**  Outcomes – Comment on the extent to which PPI influenced the study overall. Describe positive and negative effects | PPI was used to refine target behaviours, shape intervention design, and enhance the real‑world relevance of the intervention. It ensured the intervention was likely to be feasible to deliver, respectful of GP autonomy, acceptable to patients, and tailored to local the local context. PwLE refined the messaging of the intervention and the final format of patient resources. Without PPI, the intervention would likely have been more theoretically driven and less aligned with real-world primary care constraints. No negative effects were identified. |
| **5. Reflections**  Critical perspective - Comment critically on the study, reflecting on the things that went well and those that didn’t, so others can learn from this experience | - A strength of the PPI process was the inclusion of diverse stakeholders, enabling integration of multiple viewpoints across the entire intervention-development process. - Holding separate stakeholder groups for PwLE and clinician allowed for more open and nuanced discussions, though it increased time and resource requirements and limited opportunities for cross-group dialogue   Learnings from our experience:   - Following a four-step process might have constrained the extent to which PwLE and clinicians were able to shape the intervention - Involving PWLE earlier in shaping the research questions may have further enhanced co-design. - Engagement was limited to one geographic region, which may affect transferability - PwLE reflected on their experience of contributing to the study. Working with a collaborative research team, having a group of contributors with diverse views a comfortable accessible meeting space and funding to finance their time were important |
